# Pyrroloquinoline quinone (PQQ) protects mitochondrial function of HEI-OC1 cells under premature senescence

**DOI:** 10.1038/s41514-022-00083-0

**Published:** 2022-04-19

**Authors:** Ying Gao, Teru Kamogashira, Chisato Fujimoto, Shinichi Iwasaki, Tatsuya Yamasoba

**Affiliations:** 1 Department of Otolaryngology and Head and Neck Surgery, Faculty of Medicine, University of Tokyo, Tokyo, Japan; 2 Department of Otolaryngology and Head and Neck Surgery, The Second Affiliated Hospital of Xi’an Jiaotong University, Xi’an, China; 3 Department of Otolaryngology & Head and Neck Surgery, Nagoya City University Graduate School of Medicine, Nagoya, Japan

**Keywords:** Senescence, Organelles

## Abstract

The aim of this study was to investigate the effects of pyrroloquinoline quinone (PQQ), an oxidoreductase cofactor, on the H_2_O_2_-induced premature senescence model in HEI-OC1 auditory cells and to elucidate its mechanism of action in vitro. Cells were treated with PQQ for 1 day before H_2_O_2_ (100 μM) exposure. Mitochondrial respiratory capacity was damaged in this premature senescence model but was restored in cells pretreated with PQQ (0.1 nM or 1.0 nM). A decrease in mitochondrial potential, the promotion of mitochondrial fusion and the accelerated movement of mitochondria were all observed in PQQ-pretreated cells. The protein expression of sirtuin 1 (SIRT1) and peroxisome proliferator-activated receptor gamma coactivator-1α (PGC-1α) were significantly decreased under H_2_O_2_ exposure while they were increased with PQQ pretreatment, and PGC-1α acetylation was significantly decreased. In conclusion, PQQ has a protective effect on the premature senescence model of HEI-OC1 auditory cells and is associated with the SIRT1/PGC-1α signaling pathway, mitochondrial structure, and mitochondrial respiratory capacity.

## Introduction

Aging is a physiological phenomenon that occurs in all eukaryotes. Cellular senescence manifests as a stable cell arrest with active metabolism, which plays an important role in aging^1,2^. H_2_O_2_ (hydrogen peroxide) is one type of reactive oxygen species (ROS) which has been widely used to achieve oxidative stress-induced premature senescence within a short period of time^3,4^. In our previous study, short H_2_O_2_ exposure led to a significant decrease in the cell population and mitochondrial respiratory capacity and resulted in an imbalance of mitochondrial fusion/fission^5^. The regulation of aging also relies on ROS function in redox signaling, and the control of oxidative stress is essential^6^.

Pyrroloquinoline quinone (PQQ) was first reported in 1964 by Hauge et al. as a new coenzyme distinct from nicotinamide adenine dinucleotide (NADH) and flavin in glucose dehydrogenase (GDH)^7^, and its structure was determined by X-ray analysis in 1979^8,9^. PQQ is contained in fruits and vegetables, such as kiwi fruit, parsley, cocoa powder, fermented soybeans (Japanese natto) and green peppers, and in human breast milk^10–12^. PQQ is a novel biofactor of physiological importance^13^, and has been reported to be involved in a variety of biological functions that are clearly beneficial to survival, such as growth and fertility in newborns^14^. It has also been reported to protect against glutamate-induced cell apoptosis in primary cultured hippocampal neurons^15^. PQQ is especially effective in neutralizing superoxide and hydroxyl radicals^16,17^, which cause mitochondrial dysfunction, and it also antagonizes several types of oxidative stress-induced cell damage, including reoxygenation cardiac injury^18^, chronic heart failure^19^, ethanol-induced liver damage^20^, and hyperoxia-induced cognitive deficits^21^.

PQQ is also involved in the control of redox processes in the mitochondrial respirator chain^22^, the attenuation of oxidative stress in mitochondria^23,24^, and the protection of neurons^25,26^. PQQ not only protects mitochondria from oxidative stress but also promotes mitochondrial biogenesis^27^. Dietary supplementation of PQQ improves mitochondrial amounts and lipid metabolism in rats^28^ and has been shown to improve respiratory quotients by increasing the mitochondrial numbers and function in mice^29^. PQQ prevents rotenone-induced neurotoxicity in Parkinson’s disease models by promoting mitochondrial function and regulating mitochondrial fission and fusion^30^. In humans, dietary supplementation with PQQ recovers the antioxidant potential, attenuates the inflammatory response, and increases urinary metabolites related to mitochondrial functions^31^. Considering these findings, the protective effect of PQQ in mitochondrial biogenesis is also expected for inner ear cells.

Mitochondrial biogenesis is mediated by peroxisome proliferator-activated receptor γ coactivator-1α (PGC-1α) under the control of the histone deacetylase sirtuin 1 (SIRT1)^32^ which has emerged as a crucial regulator of mitochondrial function in vascular smooth muscle^33^, liver^34^, kidney^35–37^, and heart^38,39^. PQQ has positive effects on biological activities and neural functions in SK-N-SH cells^40^, HepG2 cells^41^, and in C57BL/6 mice by increasing PGC-1α expression^40^. PQQ also stimulates mitochondrial biogenesis through phosphorylation of the cAMP response element-binding protein and an increase of PGC-1α expression in mouse hepatocytes^27,42^. In humans, the supplementation of PQQ has been reported to improve peak oxygen consumption and impact mitochondrial biogenesis by elevating PGC-1α protein content^43^. The SIRT1/PGC-1α signaling pathway modulation with the administration of PQQ may also play an important role in protecting mitochondrial function in the inner ear tissues.

This study aims to assess mitochondrial metabolic activity, mitochondrial network structure, mitochondrial motility, and the SIRT1/PGC-1α signaling pathway under PQQ treatment in the immortalized Corti-derived auditory epithelial cell line HEI-OC1 using the H_2_O_2_-induced premature senescence model.

## Results

### High-concentration PQQ-induced cytotoxicity

**Fig. 1:**
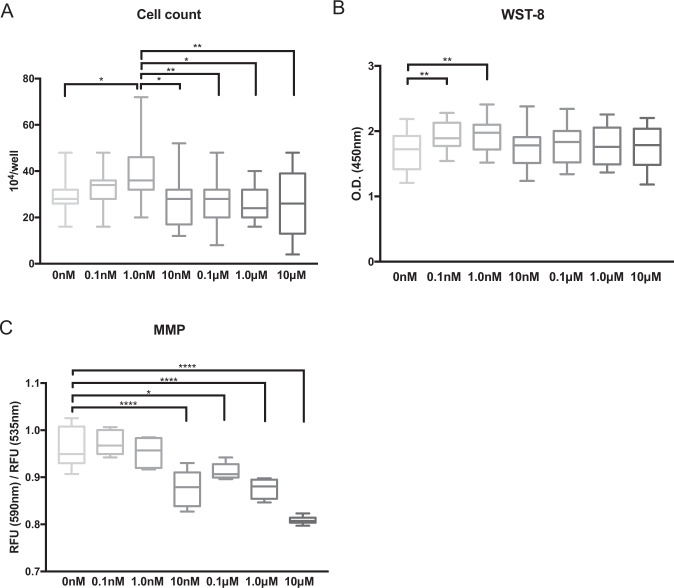
Effects of PQQ on cell proliferation, cell metabolic activity, and the mitochondrial membrane potential in HEI-OC1 cells evaluated using multiple assays. **A** Cell proliferation rate analyzed by cell counting after PQQ pretreatment for 1 day. **B** Cell metabolic activity measured with the WST-8 formazan assay after PQQ pretreatment for 1 day, shown in optical density (O.D.). The higher value indicates higher metabolic activity. **C** Mitochondrial membrane potential (MMP) measured with JC-1 fluorescence staining after PQQ pretreatment for 1 day. The ratio of red signal (relative fluorescence units (RFU) at 590 nm; mitochondrial polarized cells) to green signal (RFU at 535 nm; mitochondrial depolarized cells) is shown (*n* = 5 per group). Box plot shows statistical parameters as follows; central line: median; box limits: first and third quartile; whiskers: minimum and maximum. *P < 0.05, **P < 0.01, ****P < 0.0001.

We first evaluated the cytotoxicity of PQQ in HEI-OC1 cells to confirm the safety of PQQ as a medicine. To achieve this, we examined the cell population doubling rate, metabolic enzyme activity using WST-8 formazan, and the mitochondrial membrane potential (MMP) using the JC-1 fluorescence dye, techniques that have been previously reported for evaluating PQQ and similar compounds in cell lines^40,44^. The population doubling rate increased significantly compared to the control when the concentration of PQQ was 1.0 nM (*P* < 0.05), but it then decreased at higher concentrations; the population doubling rate was greater at 1.0 nM compared to that at concentrations greater than 10 nM (Fig. 1A) (*P* < 0.05). The metabolic enzyme activity was increased significantly compared to the control at PQQ concentrations of 0.1 nM and 1.0 nM, whereas there was no significant difference when the concentration was greater than 10 nM (Fig. 1B). The MMP decreased significantly compared to the control when the concentration was 10 nM or greater (Fig. 1C). These results indicate that, for the HEI-OC1 auditory cells, the optimum concentration of PQQ determined during the evaluation of cell metabolism was 1.0 nM and that PQQ induces cytotoxicity when applied at higher concentrations. The optimum concentration of PQQ we found was similar to those reported previously^40^, thus we decided to use PQQ concentrations of 0.1 nM and 1.0 nM in the following experiments.

### PQQ pretreatment before H_2_O_2_ exposure increased cell proliferation and cell metabolism in HEI-OC1 cells but decreased the mitochondrial membrane potential

**Fig. 2:**
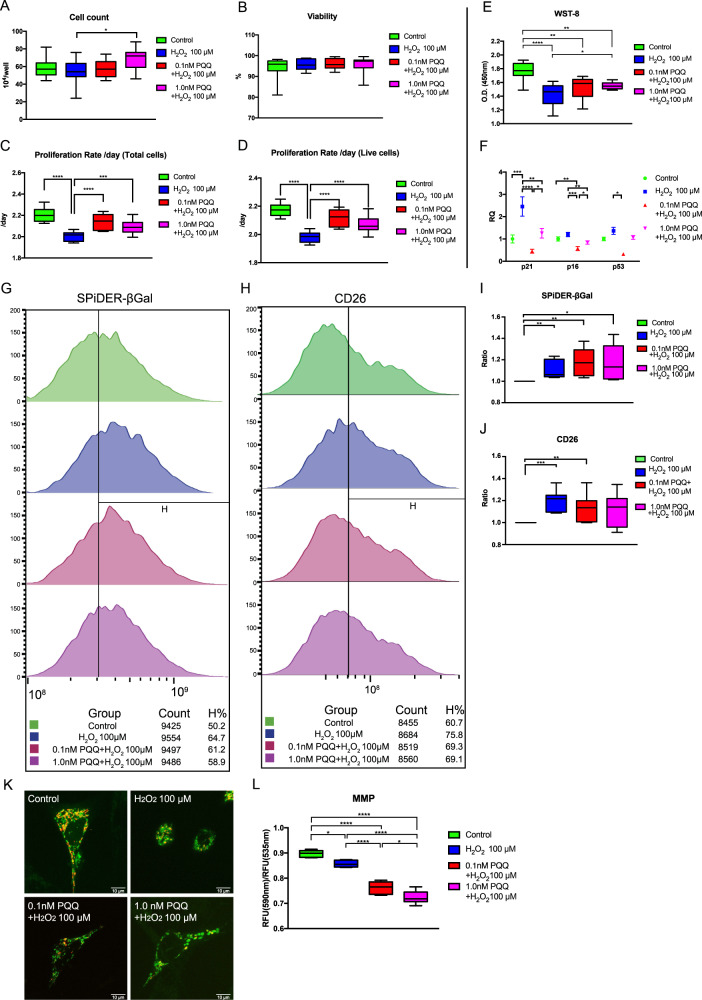
Cell proliferation, cell metabolic activity, senescence markers, and mitochondrial membrane potential (MMP) in HEI-OC1 cells with premature cellular senescence induced by H_2_O_2_ exposure with and without PQQ pretreatment. The effects of PQQ under H_2_O_2_ exposure are evaluated using multiple assays in the same manner as shown in Fig. 1. **A** Cell count 1 day after H_2_O_2_ exposure. **B** Cell viability 1 day after H_2_O_2_ exposure. **C** Cell proliferation rate using total cell count for 3 days after H_2_O_2_ exposed. **D** Cell proliferation rate using live-cell count for 3 days after H_2_O_2_ exposure. **E** Cell metabolic activity measured with WST-8 formazan assay. **F** Relative mRNA expressions of Cdkn1a (p21), Cdkn2a (p16), and Trp53 (p53) using β-actin as an internal control. RQ relative quantification. **G** The changes of senescence-associated β-galactosidase (SA-β-Gal) using SPiDER-βGal staining in flow cytometry analysis. **H** The changes of dipeptidyl peptidase DPP4 (CD26) cell surface protein marker in flow cytometry analysis. **I** The increased ratio in positivity rate over control of SPiDER-βGal positive cells. **J** The increased ratio in positivity rate over control of CD26 positive cells. **K** Imaging of mitochondria stained with JC-1. Green signal indicates low MMP and red signal indicates high MMP. Scale bar, 10 µm. **L** MMP measured with JC-1. (**A**–**D**, **L**: *n* = 6 per group, **E**, **I**, **J**: *n* = 5 per group, **F**: *n* = 3 per group). Box plot shows statistical parameters as follows; central line: median; box limits: first and third quartile; whiskers: minimum and maximum. RQ data are shown as mean ± standard deviation. *P < 0.05, **P < 0.01, ***P < 0.001, ****P < 0.0001.

H_2_O_2_ is widely utilized as an agent to induce premature cellular senescence in cell lines^45–47^ including HEI-OC1 cells^48^. We have previously shown that premature cellular senescence was induced in HEI-OC1 cells by exposing them to H_2_O_2_ at a concentration of 100 μM for 1 h^5^. We evaluated the protective effect of pretreatments of 0.1 nM and 1.0 nM PQQ for 1 day on auditory cells with H_2_O_2_-induced premature senescence using population doubling rate analysis and metabolic enzyme activity analysis. The cell counts significantly increased in H_2_O_2_-exposed cells pretreated with 1.0 mM PQQ, although they remained unchanged in the other conditions (Fig. 2A). There was no significant decrease of viability in H_2_O_2_-exposed cells pretreated with PQQ (0.1 mM and 1.0 mM) (Fig. 2B). The population doubling rate decreased significantly in the H_2_O_2_-exposed group (with no PQQ pretreatment), whereas pretreatment with 0.1 nM or 1.0 nM PQQ significantly ameliorated the decrease induced by H_2_O_2_ exposure (Fig. 2C, D). These results indicate that H_2_O_2_ exposure induced premature senescence in HEI-OC1 cells and PQQ pretreatment had a protective effect against H_2_O_2_ exposure. H_2_O_2_ exposure decreased the metabolic activity of HEI-OC1 cells even when they were pretreated with PQQ, but the decrease in metabolic activity was significantly ameliorated when the cells were pretreated with 1.0 nM PQQ (Fig. 2E).

We also evaluated the expression of cell cycle regulators (Cdkn1a (p21^WAF1/CIP1^), Cdkn2a (p16^INK4A^), and Trp53 (p53)), senescence-associated β-galactosidase (SA-β-Gal), and the dipeptidyl peptidase DPP4 (CD26) cell surface protein as cellular senescence biomarkers. The expression of the primary senescence markers p21, p16, and p53 are associated with cell cycle arrest in mediating senescence and the senescent phenotype^49,50^. SA-β-Gal is widely utilized as a senescence marker in aging research^51–53^, and has also been previously evaluated in HEI-OC1 cells^2,54^. CD26 is recently reported as a senescence marker^55,56^, and is emerging as a therapeutic target^57^. While H_2_O_2_ exposure increased the expression of p21 and the activity of SA-β-Gal and CD26, the expression of p21, p16, and p53 were all alleviated by 0.1 nM PQQ pretreatment (Fig. 2F–J). These results also support the theory that H_2_O_2_ exposure induces premature senescence and that PQQ pretreatment protects HEI-OC1 cells from H_2_O_2_-induced premature senescence.

The MMP was decreased by exposure to H_2_O_2_ and more significantly decreased when the HEI-OC1 cells were pretreated with PQQ (Fig. 2K, L). These results suggest that PQQ pretreatment protects HEI-OC1 cells from H_2_O_2_-induced premature senescence and that PQQ has a mitochondrial uncoupling effect. The mitochondrial reactive oxygen species (ROS) evaluated with MitoSOX Red was not decreased by exposure to H_2_O_2_ whereas PQQ pretreatment significantly increased the ROS level (Supplementary Data). These results indicate that PQQ mildly increases the ROS levels, which can help to maintain the regulation of ROS levels in HEI-OC1 cells and so protect against premature senescence.

### PQQ alleviated ultrastructural damage of mitochondria induced by exposure to H_2_O_2_ in HEI-OC1 cells

**Fig. 3:**
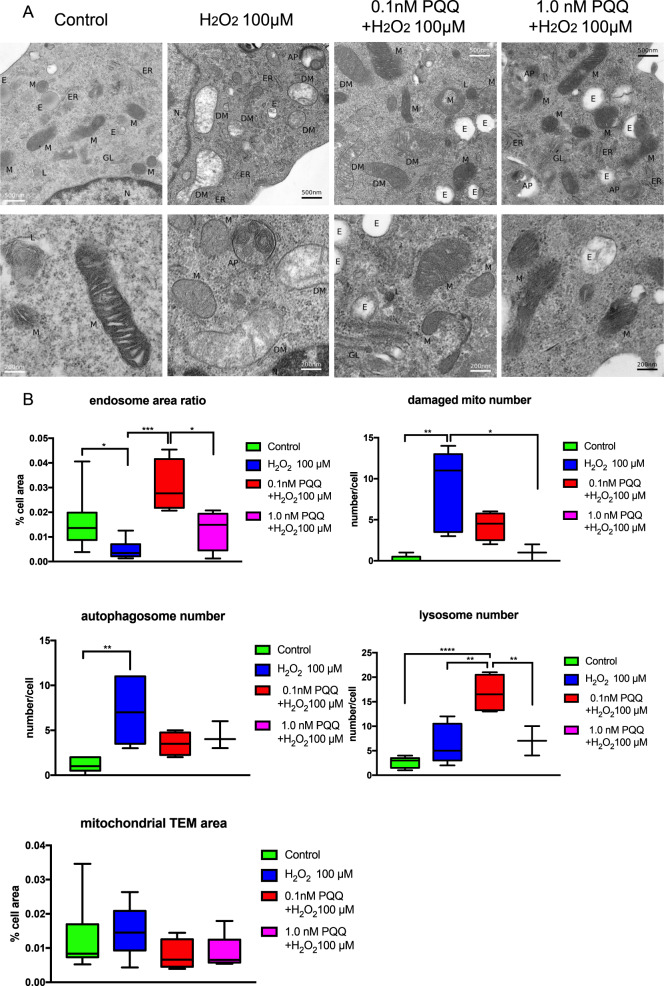
Mitochondrial ultrastructure in HEI-OC1 cells exposed to H_2_O_2_ (100 µM) with and without PQQ pretreatments (0.1 nM and 1.0 nM), and control cells. **A** Short exposure to H_2_O_2_ induces fine structural damage to mitochondria, which is alleviated with PQQ pretreatment. From left to right: control cells, cells exposed to H_2_O_2_, cells exposed to H_2_O_2_ after 0.1 nM PQQ pretreatment, and cells exposed to H_2_O_2_ after 1.0 nM PQQ pretreatment. AP autophagosome, DM damaged mitochondria, E endosome, ER endoplasmic reticulum, GL Golgi body, L lysosome, M mitochondria, N nucleus. Scale bar, 500 nm in top row, 200 nm bottom row. **B** The numerical analysis of TEM images. The endosome ratio, damaged mitochondria number, autophagosome number, and lysosome number were calculated by visual inspection (*n* = 5 per group). Box plot shows statistical parameters as follows; central line: median; box limits: first and third quartile; whiskers: minimum and maximum. *P < 0.05, **P < 0.01, ***P < 0.001, ****P < 0.0001.

We also evaluated the protective effect of PQQ pretreatment on auditory cells with H_2_O_2_-induced premature senescence using transmission electron microscopy (TEM) image analysis. The shape of the mitochondria under TEM in the control cells was normal, but the mitochondria in the H_2_O_2_-exposed cells showed ultrastructural damage, including typical matrix swelling, the loss of cristae in the majority of mitochondria, and the increased formation of autophagosomes (Fig. 3A). The mitochondrial damage was alleviated by PQQ pretreatment before H_2_O_2_ exposure; PQQ pretreatment improved the internal heterogeneity and the swollen shape of the mitochondria and increased the number of endosomes. Image analysis of the area of endosomes showed that the increase in the number of endosomes and lysosomes was significant in the 0.1 nM PQQ-pretreated group compared to the H_2_O_2_-exposed group with no PQQ pretreatment (Fig. 3B). In contrast, the number of autophagosomes was significantly increased in the H_2_O_2_-exposed group, whereas PQQ pretreatment suppressed this increase. These morphological findings indicate that PQQ alleviates ultrastructural damage to the mitochondria of HEI-OC1 cells induced by a short exposure to H_2_O_2_. In addition, PQQ pretreatment increased the number of both endosomes and lysosomes, which implies an acceleration of autophagy or the inhibition of lysosome function (Fig. 3B).

The area of total mitochondria including healthy mitochondria and damaged mitochondria showed no significant difference between groups (Fig. 3B). The copy numbers of the mitochondrial DNA did not show a significant difference between groups (Supplementary Data), which indicated that the changes under the exposure to H_2_O_2_ and the treatment of PQQ mainly causes morphological and structural changes in mitochondria but not the proliferation of mitochondria in the timeframe of this study. These results indicate that H_2_O_2_ exposure or PQQ pretreatment does not alter mitochondrial proliferation but causes damages or protection in mitochondrial structure.

### PQQ protected against the decline of mitochondrial respiratory capacity in the oxidative stress-induced premature senescence of HEI-OC1 cells

**Fig. 4:**
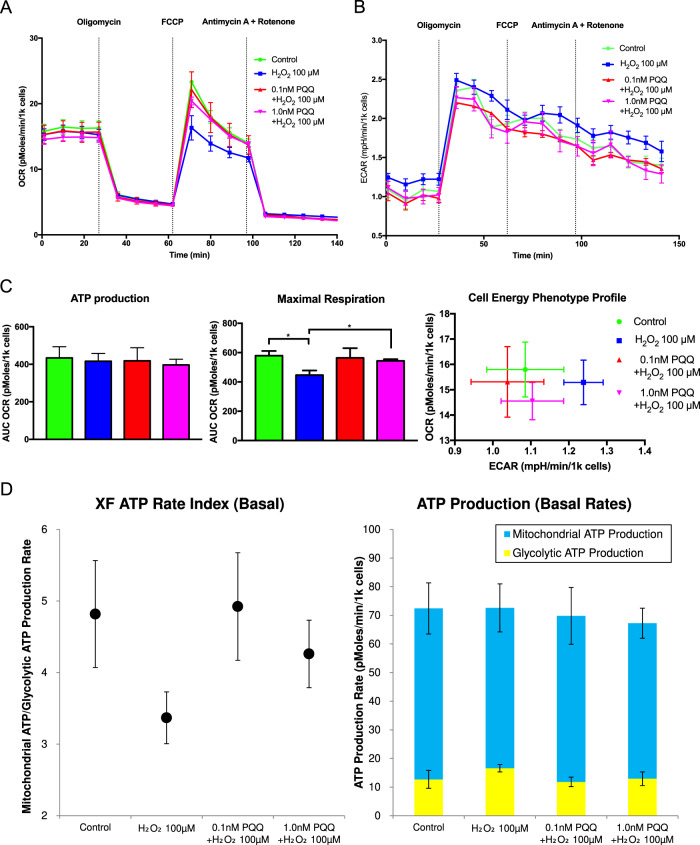
Mitochondrial biogenesis in the premature cellular senescence model with and without PQQ treatment. The effects of PQQ pretreatment in HEI-OC1 cells exposed to H_2_O_2_ were evaluated using a XF24 flux analyzer. **A** Oxygen consumption rate (OCR) diagram of the XF assay, normalized by the number of cells. **B** Extracellular acidification rate (ECAR) diagram of the XF assay. **C** ATP production rate quantified with OCR measured following the injection of oligomycin, maximal respiration rates measured following injection of FCCP, and cell energy phenotype profile of the XF assay. **D** Basal ATP production rate and ATP rate index of the XF assay (*n* = 5 per group). Data are shown as mean ± standard deviation. *P < 0.05.

In order to confirm the protective effect of PQQ pretreatment in mitochondrial biogenesis and metabolic function in HEI-OC1 cells, we evaluated the oxygen consumption rate (OCR) and the extracellular acidification rate (ECAR) using a XF24 extracellular flux analyzer, which measures the fundamental parameters of the electron transport chain (ETC), basal OCR, ATP-linked respiration, maximal OCR, spare respiratory capacity and proton leak (Fig. 4A, B). The basal ATP production speed did not differ significantly between groups; however, the maximal respiration measured following the injection of a mitochondrial uncoupling agent (carbonyl cyanide-4-(trifluoromethoxy) phenylhydrazone (FCCP)) decreased significantly in the H_2_O_2_-exposed group compared to the control group, while it was protected significantly in both the 0.1 nM and 1.0 nM PQQ-pretreated groups compared to the H_2_O_2_-exposed group (Fig. 4C). ECAR tended to increase only in the H_2_O_2_-exposed group, although the change was not significant (Fig. 4B, D). ATP production speed represented by OCR, ECAR, and phosphate/oxygen ratio (P/O ratio) showed a slight increase of glycolytic ATP production under H_2_O_2_ exposure which was alleviated by PQQ pretreatment, although the difference was not significant between groups (Fig. 4D). These results indicate that PQQ prevents the decline of mitochondrial respiratory capacity under H_2_O_2_ exposure in HEI-OC1 cells.

**Fig. 5:**
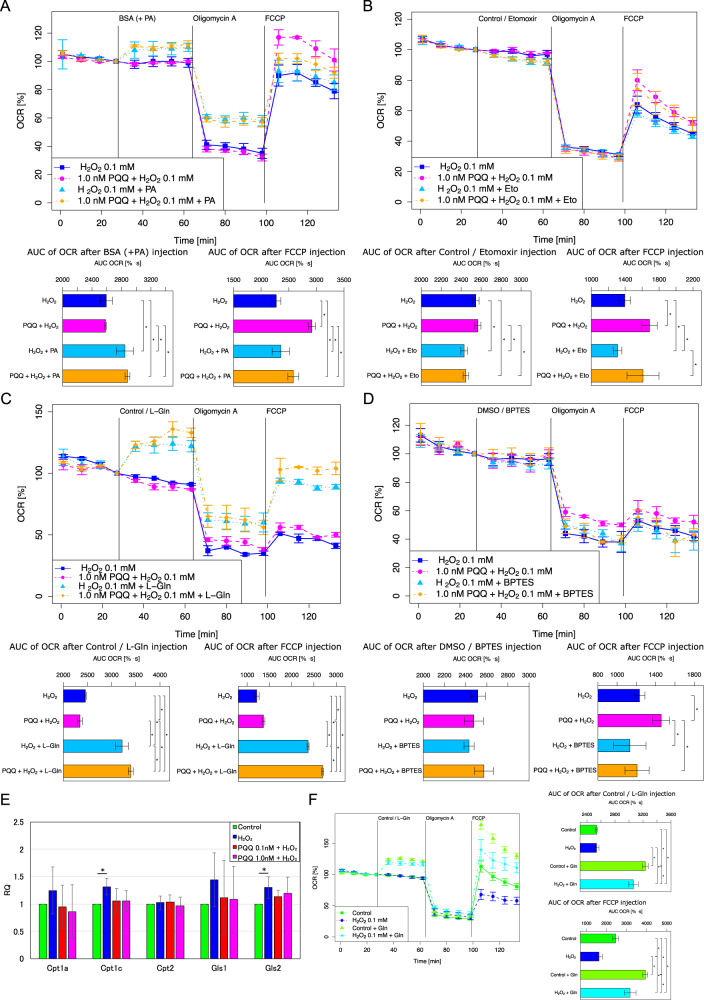
Substrate-specific mitochondrial biogenesis in the premature cellular senescence model with and without PQQ treatment. The substrate-specific effects of PQQ pretreatment in HEI-OC1 cells exposed to H_2_O_2_ were evaluated using a XF24 flux analyzer; **A** Oxygen consumption rate (OCR) under palmitic acid supplementation. **B** OCR under Cpt (Carnitine palmitoyltransferase) inhibition by Etomoxir. **C** OCR under glutamine supplementation. **D** OCR under Gls (Glutaminase) inhibition by BPTES ((bis-2-(5-phenylacetamido-1,3,4-thiadiazol-2-yl)ethyl sulfide). **E** Relative mRNA expressions of Cpt1a, Cpt1c, Cpt2, Gls1, and Gls2 using β-actin as an internal control. **F** OCR under glutamine supplementation in HEI-OC1 cells in the control group or H_2_O_2_-exposed group (*n* = 5 per group). Data are shown as mean ± standard deviation. *P < 0.05.

The maximal respiration after the injection of a mitochondrial uncoupling agent indicates the mitochondrial respiratory capacity or reserve capacity, which works effectively in response to high-energy demand or oxidative stress^58,59^. The mechanism of improvement in respiratory capacity mainly concerns the ETC because the medium in this assay contains glucose and pyruvic acid, but not glutamate or fatty acid (Supplementary Information). To further confirm the substrates on which HEI-OC1 cells depend, the metabolism regarding fatty acid oxidation (FAO) and glutaminolysis were analyzed. The palmitic acid supplementation increased the OCR whereas respiratory capacity was decreased; indicating that fatty acid worked as an uncoupler rather than an oxidation substrate, and fatty acid was not mainly utilized in HEI-OC1 cells (Fig. 5A). Carnitine palmitoyltransferase (CPT) inhibitor, Etomoxir, decreased respiratory capacity without PQQ pretreatment, but the decrease was not significantly different compared to that with PQQ treatment, indicating that FAO was not the main pathway in the recovery of respiratory capacity (Fig. 5B). Glutamine supplementation increased the OCR and also respiratory capacity, although the protective effect of PQQ was still present under glutamine supplementation (Fig. 5C). Glutaminase (GLS) inhibitor, (bis-2-(5-phenylacetamido-1,3,4-thiadiazol-2-yl)ethyl sulfide (BPTES), disabled the protective effect by PQQ pretreatment (Fig. 5D), indicating that glutaminolysis is one of the alternative substrate pathways to protect damaged mitochondrial in HEI-OC1 cells. Other mechanisms including mitochondrial structural changes, however, may underlie in respiratory capacity recovery effect with PQQ pretreatment, because glutamine supplementation did not fully recover respiratory capacity. The expression of CPT1C (Carnitine palmitoyltransferase 1C) was increased significantly under the exposure to H_2_O_2_ compared to the control group, which indicates that the energy metabolism was promoted to FAO from main glucose metabolism under the exposure to H_2_O_2_, whereas these expressions were nearly completely recovered to control level by PQQ pretreatment (Fig. 5E). The expression of glutaminase 2 (GLS2) was also increased significantly under the exposure to H_2_O_2_ compared to the control group, but the increase of GLS2 under PQQ pretreatment did not significantly different compared to the control group (Fig. 5E). The increase in GLS2 expression did not result in an increase in OCR under glutamine treatment, suggesting that the increase in GLS2 expression may indicate stress to the cells (Fig. 5F). These results indicate that the metabolic shift from glucose substrate-dependent pathway to FAO or glutaminolysis was induced under the exposure to H_2_O_2_ regarding the mitochondrial damage, whereas the promotion was not enough to compensate respiratory capacity decrease and was rather a marker of H_2_O_2_-exposed stress, and this shift was held to the nearly normal state with PQQ pretreatment protection.

### PQQ has the potential to protect against morphological and dynamic deteriorations in HEI-OC1 cells with short exposures to H_2_O_2_

**Fig. 6:**
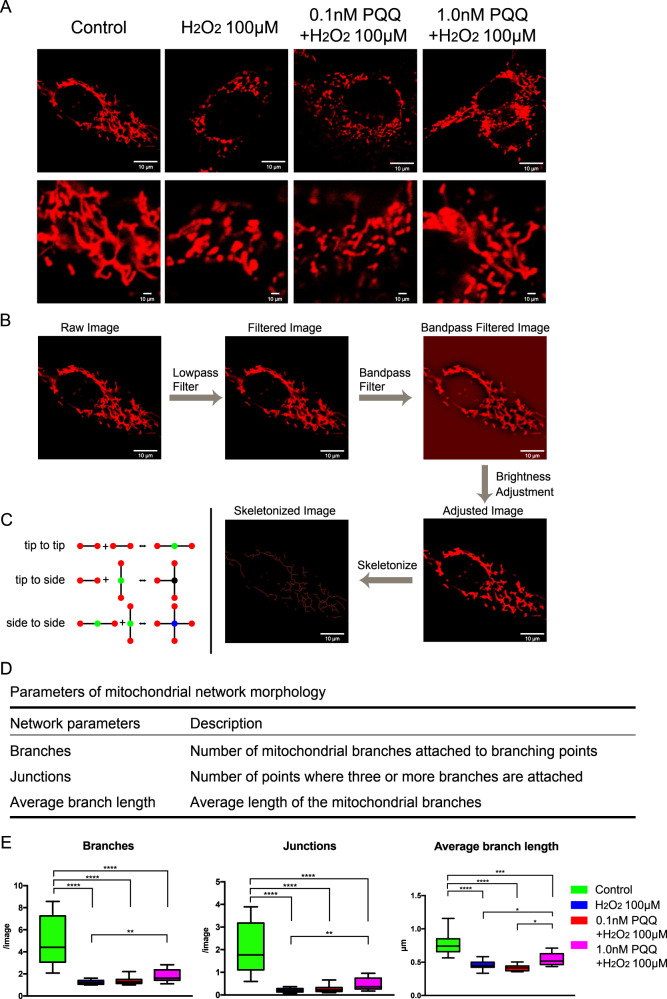
The morphological evaluation of mitochondria in the premature cellular senescence model under PQQ pretreatment. The morphological changes with PQQ pretreatment in HEI-OC1 cells exposed to H_2_O_2_ to induce premature cellular senescence were evaluated using microscopic imaging. **A** Mitochondrial images stained with MitoTracker Orange CMTMRos. Scale bar, 10 µm. **B** The image processing flow of the skeletonized image analysis. **C** The different types of dynamic mitochondrial regulations including fusion and fission mechanisms involving network nodes (tip-to-tip, tip-to-side, or side-to-side). Graph representation of the mitochondrial reticulum using four node types: 1 (magenta), 2 (green), 3 (dark blue), and 4 (blue). **D** The descriptions of parameters in the mitochondrial network morphology. **E** The number of branches, junctions, and the average length of branches in each image (**E**: *n* = 5 per group; repeated experiments). Box plot shows statistical parameters as follows; central line: median; box limits: first and third quartile; whiskers: minimum and maximum. *P < 0.05, **P < 0.01, ***P < 0.001, ****P < 0.0001.

We evaluated the structure and dynamic motility of the mitochondria to confirm the improvements in mitochondrial function under PQQ pretreatment. The HEI-OC1 cells were stained with TMRE fluorescence dye, which accumulates in mitochondria due to the MMP, and were observed under confocal microscopy. The early alterations in mitochondrial shape and motility represent the dynamic function of mitochondria in auditory cells^5^. The control group had a good balance of mitochondrial fusion and fission, while fragmented mitochondria with a balloon-like shape or hyperfused mitochondria with an enlarged shape were observed in the H_2_O_2_-exposed group (Fig. 6A). In the 0.1 nM or 1.0 nM PQQ-pretreated groups, the mitochondrial shapes had partly recovered to the normal balance (Fig. 6A). In order to quantify the recovery of the mitochondrial structures, we processed the mitochondrial images and analyzed the specific parameters of the mitochondrial network nodes (Fig. 6B)^5^. The mitochondrial dynamics include different types of fusion and fission mechanisms of the network nodes (tip-to-tip, tip-to-side, or side-to-side) (Fig. 6C), and the specific parameters show the condition of mitochondrial dynamics (Fig. 6D). The number of branches, the number of junctions and the average branch length all increased significantly in 0.1 nM and 1.0 nM PQQ-pretreated groups compared to the H_2_O_2_-exposed group; however, mitochondrial dynamics biased toward the fusion process was observed in the 0.1 nM or 1.0 nM PQQ-pretreated groups (Fig. 6E). These results indicate that PQQ pretreatment assists in the recovery from morphological damage caused to the mitochondria by H_2_O_2_ exposure and also aids the recovery of mitochondrial dynamics in HEI-OC1 cells.

**Fig. 7:**
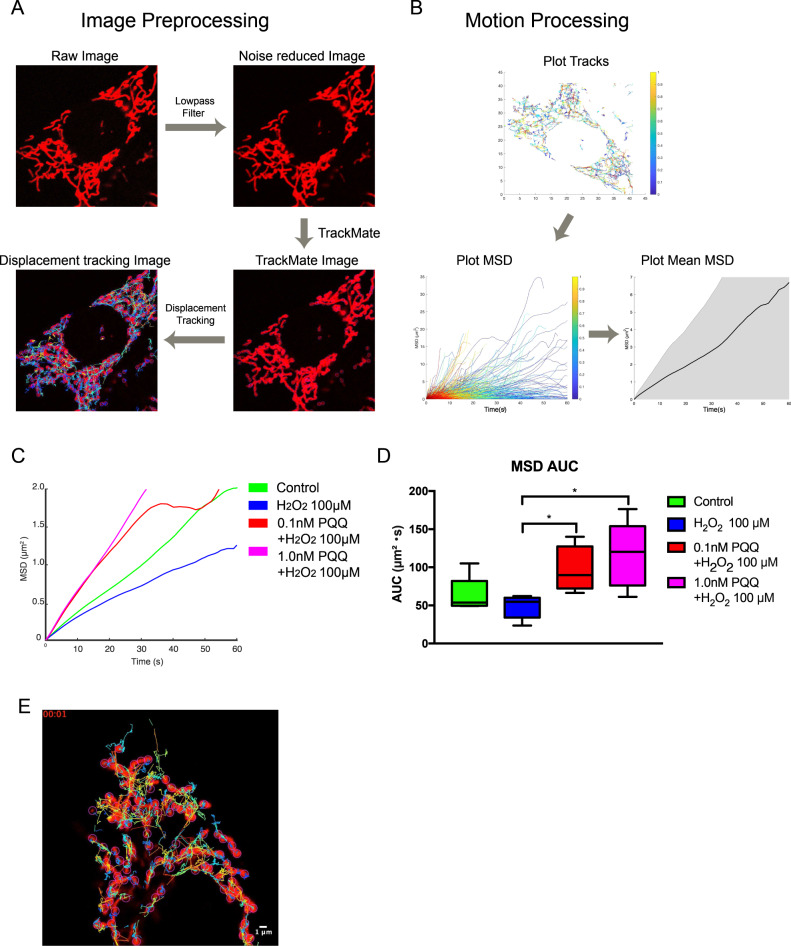
The dynamic evaluation of mitochondrial motility in the premature cellular senescence model under PQQ pretreatment. The changes in mitochondrial dynamics with PQQ pretreatment in HEI-OC1 cells exposed to H_2_O_2_ to induce premature cellular senescence were evaluated using microscopic imaging and image processing. **A** Image preprocessing flow of the motility analysis. **B** Motility analysis of the mitochondria. **C** The average of the mean square displacement (MSD) of each group. **D** The area under curve (AUC) of the MSD of each group. **E** Mitochondrial motion tracking video. The mitochondrial particle tracking lasted for 3 min in 3 s of intervals under ImageJ (**D**: *n* = 5 per group; repeated experiments). Box plot shows statistical parameters as follows; central line: median; box limits: first and third quartile; whiskers: minimum and maximum. *P < 0.05.

For the analysis of mitochondrial motility, particle tracking was derived from spatiotemporal image sequences, and then the kinetics of the moving particles were evaluated quantitatively using the TrackMate plugin in ImageJ/FIJI and @msdanalyzer on the MATLAB platform (Fig. 7A, B, E). The mean square displacement (MSD) was calculated from the tracking of the mitochondria, and the area under the curve (AUC) of the MSD plot of each group is shown in Fig. 7C, D. The mitochondrial motility in the short-exposure H_2_O_2_ group tended to decrease compared to the control group, while it increased in the PQQ-pretreated groups. The AUC of the MSD plot showed significant increases in the PQQ-pretreated groups compared to the short-exposure H_2_O_2_ group (Fig. 7D). These results also support our proposal that PQQ pretreatment protects mitochondrial dynamics under H_2_O_2_ exposure in HEI-OC1 cells.

### Pretreatment with PQQ increased the expression of SIRT1 and PGC-1α in H_2_O_2_ short exposure HEI-OC1 cells, inducing deacetylation of PGC-1α

**Fig. 8:**
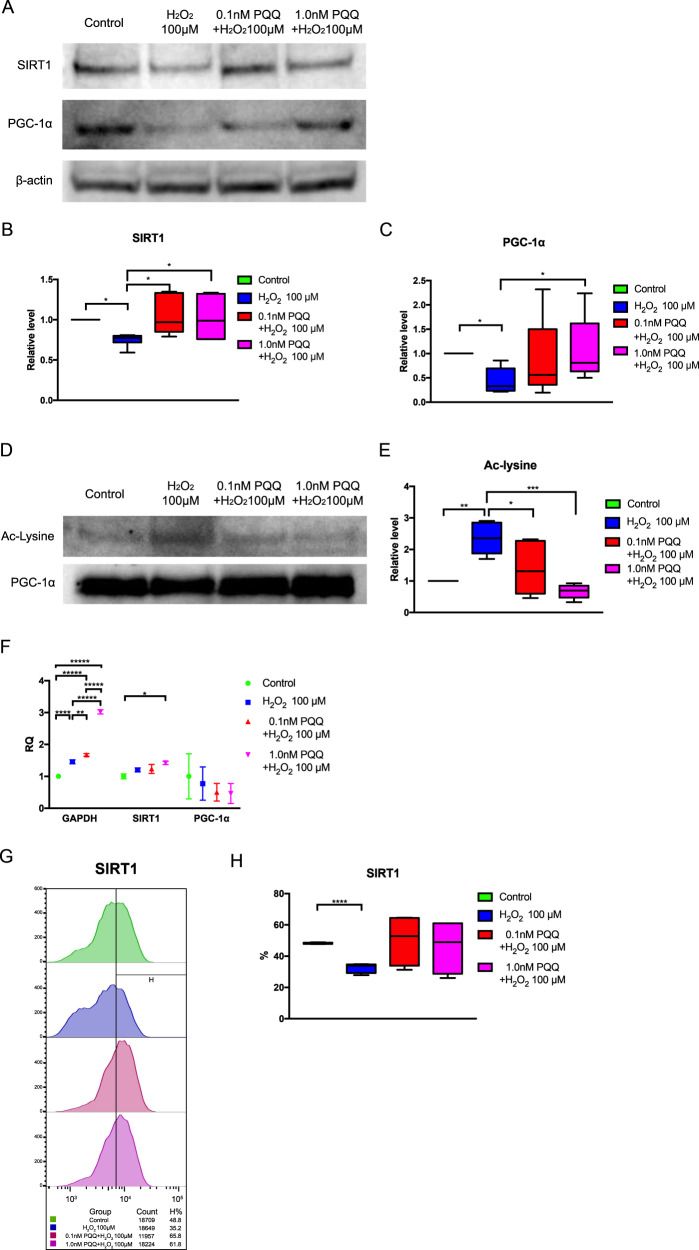
Analysis of protein expression and protein acetylation in the premature cellular senescence model under PQQ pretreatment. **A** SIRT1 and PGC-1α protein expression was analyzed by Western blotting (upper, middle) using β-actin as a loading control (lower). **B** Relative protein expression level of SIRT1 normalized by β-actin. *n* = 10 per group. **C** Relative protein expression level of PGC-1α normalized by β-actin. **D** Acetylation of PGC-1α analyzed by immunoprecipitation. **E** Relative acetylation level of PGC-1α normalized by PGC-1α. **F** Relative mRNA expressions of GAPDH, SIRT1, and PGC-1α using β-actin as an internal control. **G** The changes of SIRT1 expression in flow cytometry analysis. **H** The rate of SIRT1 positive cells (**B**, **C**, **E**, **H**: *n* = 5 per group, **F**: *n* = 3 per group; repeated experiments). Box plot shows statistical parameters as follows; central line: median; box limits: first and third quartile; whiskers: minimum and maximum. RQ data are shown as mean ± standard deviation. *P < 0.05, **P < 0.01, ***P < 0.001, ****P < 0.0001, *****P < 0.00001.

To explore the pathways by which PQQ pretreatment led to the improvement of mitochondrial function, we investigated changes in the expression of SIRT1 and PGC-1α involving the regulation of mitochondrial metabolism. The expression of SIRT1 and PGC-1α decreased significantly in the H_2_O_2_ short-exposure group, while it recovered significantly in the 1.0 nM PQQ-pretreated group (Fig. 8A–C, G). In addition, the acetylation of PGC-1α increased significantly in the H_2_O_2_ short-exposure group, while it attenuated significantly in the 1.0 nM PQQ-pretreated group (Fig. 8D, E). No significant change was observed in the mRNA expression levels of SIRT1 and PGC-1α and there was no correlation seen between the protein and mRNA expression (Fig. 8F). These results indicate that other post-translational modification factors including microRNA (miRNA) might regulate SIRT1 and PGC-1α at the transcriptional level^60–63^. These results indicate that H_2_O_2_ short exposure decreases the protein expression of SIRT1 and PGC-1α, while PQQ pretreatment increases the protein expression of SIRT1 and PGC-1α under H_2_O_2_ short exposure in HEI-OC1 cells, inducing the deacetylation of PGC-1α.

## Discussion

**Fig. 9:**
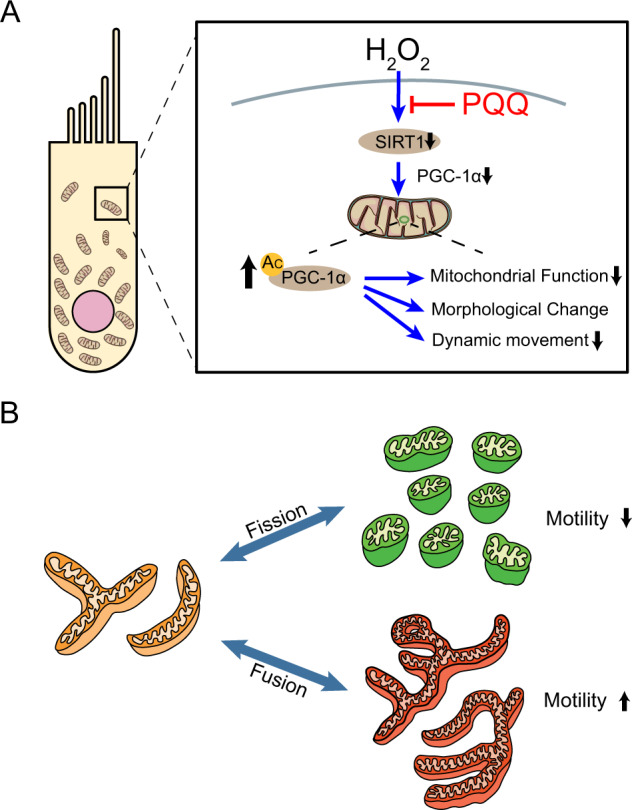
Schematic diagram of the mechanism of HEI-OC1 mitochondrial biogenesis modulation under H_2_O_2_ exposure and PQQ protection. **A** Sirtuin 1 (SIRT1) expression and deacetylation activity are decreased in HEI-OC1 cells by H_2_O_2_ exposure. PGC-1α mediates the protective effect of SIRT1 expression and mitochondrial biogenesis and function. PQQ improves mitochondrial biogenesis and function by restoring SIRT1 expression and deacetylation activity under H_2_O_2_ exposure in HEI-OC1 cells. **B** Schematic diagram of mitochondrial morphological changes.

In this study, we demonstrated that PQQ has the potential to protect against oxidative stress-induced premature cellular senescence in auditory cells by enabling the recovery of mitochondrial function by restoring the regulation of SIRT1/PGC-1α, the protein expression of SIRT1, and the deacetylation of PGC-1α, and by facilitating the recovery of mitochondrial biogenesis including the ATP production rate and maximum respiration rate. Restored SIRT1 leads to the mediation of PGC-1α and mitochondrial biogenesis in the auditory cells with oxidative stress-induced premature senescence (Fig. 9). To our knowledge, this is the first report which indicates the effect of PQQ in protecting against the oxidative stress-induced premature cellular senescence of auditory cells.

First, we confirmed the mitochondrial morphology and dynamics in auditory cells for evaluating the effect of PQQ pretreatment because these two changes represent the condition of the mitochondrial quality control system^64^, and previous reports indicate that PQQ improves reproduction, neonatal development, and mitochondrial function in rats by mechanisms that involve mitochondrial-related signaling pathways^28^. The acceleration of autophagy function was indicated in the PQQ-pretreated groups by TEM images. In addition, the increase of the fusion of autophagosome and lysosome in the degradation step of autophagy, leading to the formation of autolysosome, was also presumed by TEM image analysis. PQQ could ameliorate autophagy-dependent apoptosis via the lysosome-mitochondria axis in vascular endothelial cells^65^, but because these functions interact closely with each other, it is difficult to precisely separate them and evaluate their functions, so further research is needed. In addition, we focused on mitochondrial motility in auditory cells because it decreases with age in vascular smooth muscle at the same time as mitochondrial dysfunction^66^. In this study, we confirmed the improvement of mitochondrial morphology as well as mitochondrial motility with PQQ pretreatment in the oxidative stress-induced premature senescence of auditory cells, which supports the protective aspect of PQQ in the mitochondrial quality control system.

Mitochondrial function is dependent on mitochondrial structure and mitochondrial dynamics, including fission, fusion, and motility^67^. The mitochondrial transport to discrete subcellular regions may contribute to the accumulation of reactive oxygen species (ROS) in the nucleus, and the oxidative-based signaling function^68^. The fusion of mitochondria is dependent on the mitochondrial inner membrane potential and independent of microtubules or actin in neurons, and it directly reflects the mitochondrial condition^69^. The anterograde mitochondrial transport delivers healthy mitochondria to peripheral sites, while the retrograde mitochondrial transport returns damaged mitochondria to central sites, maintaining mitochondrial quality control, and the mitochondrial trafficking is crucial for neural survival^70^. Molecular pathways that control mitochondrial movement can be reduced to their effects on the balance of forces that act on mitochondria, driving and opposing movement^71^. The immobilization of mitochondria to microtubules is mediated by syntaphilin, an axonal molecule that facilitates the increase in mitochondrial volume in demyelinated axons, facilitating their survival and protecting against axonal degeneration in the central nervous system^72^. Mitochondrial motility and dynamics both regulate and reflect mitochondrial function in auditory cells^5^. The morphological and dynamic deteriorations caused by H_2_O_2_ exposure were alleviated by PQQ pretreatment in HEI-OC1 cells, which indicates that PQQ works protectively in terms of the mitochondrial function and the mitochondrial condition. The recovery of mitochondrial morphology and dynamics is the first step in the protection of mitochondrial metabolic activity in auditory cells.

The increase of expressions of FAO and glutaminolysis was observed under the exposure to H_2_O_2_, which was not enough to recover mitochondrial biogenesis, and this increase was reverted to nearly normal state by PQQ pretreatment. The induction of FAO or glutaminolysis is reported to be one of the phenotype of cellular senescence; CPT1A is overexpressed in senescent placenta-derived mesenchymal stem cells, the inhibition of CPT1A induced a change of energy metabolism and reversed senescence^73^, and GLS1 was identified as an essential gene for the survival of senescent cells^56^. The increase of expressions in FAO or glutaminolysis-related genes can draw the condition of H_2_O_2_-induced premature cellular senescence. This increase was inhibited by PQQ pretreatment, which indicates that PQQ have the protective function on mitochondria. Glutaminolysis was one of the alternative substrate pathways to protect damaged mitochondrial in HEI-OC1 cells under PQQ pretreatment, although other mechanisms including structural changes of mitochondria may underlie in respiratory capacity recovery effect with PQQ pretreatment. It has been reported that changes in mitochondrial morphology are related to the respiratory capacity of mitochondria^74^, indicating that mitochondrial structure is an important aspect of respiratory capacity protection. The mitochondrial respiratory capacity or reserve capacity respond to high-energy demand or oxidative stress and acts as a buffer to protect the cells^58,59^.

The speed of glucose consumption varies between cell lines, and the drift of the OCR in this timeframe is also observed in normal condition, indicating that the metabolism of HEI-OC1 cells is very high to decrease the glucose concentration and OCR in this timeframe. The glucose concentration in the DMEM medium during the culture of HEI-OC1 cells decreases from the initial 450 mg/dL to about 250 mg/dL within 5–6 days (Supplementary Information). This corresponds to a decrease of about 50% in 120 h and about 2% in 4 h. During the analysis of XF24, where the medium volume is very low, this decrease in glucose concentration and OCR becomes more prominent and is likely to lead to a further decrease in OCR over time during the analysis timeframe.

Importantly, the PQQ pretreatment led to the restoration of the expression of SIRT1 and PGC-1α and the deacetylation of PGC-1α in the oxidative stress-induced premature senescence of auditory cells. These results suggest that PQQ regulates mitochondrial homeostasis via SIRT1 and the deacetylation of PGC-1α in auditory cells, preventing premature senescence^75^. Recent studies suggest that PQQ activates SIRT1 and SIRT3 genes and increases NAD + activity in the human hepatocyte cell line HepG2^41^, induces deacetylation of PGC-1α and enhancement of mitochondrial activity in NIH/3T3 cells after treatment with a SIRT1 selective inhibitor^76^, and protects skeletal muscles from denervation-induced atrophy by activating PGC-1α and improving the energy metabolic profile in C57BL6/J mice^77^. These effects of PQQ activating the SIRT1/PGC-1α signaling pathway in various cells support our data, including the protective effect of PQQ for the inner ear. In addition, the age-related decrease in the expression of SIRT1 and PGC-1α in cochlear tissue is observed in C57BL/6 mice, and the overexpression of SIRT1 suppressed apoptosis and promoted cell proliferation in HEI-OC1 cells^78^. The activation of PGC-1α promoted mitochondrial biogenesis and protected them against cisplatin (CDDP)-induced ototoxicity in HEI-OC1 cells^79^. Even caloric restriction, which has an anti-aging effect, induces SIRT1 expression and improves PGC-1α expression, which can regulate energy homeostasis and extend lifespan^80^. The mRNA expression levels of SIRT1 and PGC-1α did not correlate with the protein expression levels in this study, indicating post-translational modification factors in the protective pathway of PQQ pretreatment. We consider that the expression of SIRT1 might be also regulated by microRNA (miRNA) in auditory cells, based on the results of previous studies^60–63^.

In this study, we confirmed that PQQ pretreatment had a mitochondrial uncoupling effect (the MMP decrease) in the high-concentration condition, and it increased cell viability and worked protectively under H_2_O_2_ oxidative stress (Fig. 3C, D). Indeed, the decrease of MMP by PQQ has been reported in tumor cell lines at high doses of over 15 μM^44^. The optimum concentration analysis is crucial in evaluating the protective effect of PQQ, and from the aspect of the drug screening targeting the mitochondrial protection the mitochondrial uncoupling effect is one of the key factors in reducing damage and protecting the mitochondria in relation to ROS. We selected the optimum concentration of PQQ in this study by determining the concentration at which there was no decrease in cell numbers and no decrease in mitochondrial membrane potential. In a study evaluating the cardioprotective effect of FCCP (a mitochondrial uncoupler) in the post-ischemic functional recovery of rat hearts, the optimum concentration was also determined based on the mitochondrial oxidation without mitochondrial membrane depolarization^81^, and the protective pathway was ROS-dependent^82^. Generally, the MMP shows the condition of the mitochondria, and low membrane potential is normally considered to reflect the damaged condition; however, the MMP uncoupling effect of compounds should be interpreted carefully. The mitochondrial uncouplers including intrinsic uncoupling proteins, natural uncoupling compounds or newly developed agents which are reported to have a protective effect in mitochondria against ROS damage^83^. UCP2 and UCP3, homologues of the endogenous mitochondrial uncouplers expressed in the heart, also protect against mitochondrial oxidative damage induced with cardiac ischemia-reperfusion by reducing the production of ROS^84^. FCCP reduces brain edema, decreases neuroinflammation, and improves neurological deficits following intracerebral hemorrhage by activating AMPK^85^. The newly discovered agent Ppc-1 functions as a mitochondrial uncoupler, and stimulates adipocytes to release fatty acids, thus acting as an anti-obesity agent^86,87^. Based on these previous studies, the higher respiratory flux leads to consumption of the membrane potential component of the proton motive force by ATP synthase which leads to the generation of ATP producing a mitochondrial membrane depolarization which can be one of the key factors underlying the protective mechanisms of PQQ under H_2_O_2_ exposure. Elevated mitochondrial ROS production promotes insulin resistance and obesity^88^, and the anti-obesity effect is also important in considering ROS protective agents. Low concentration of a mitochondrial uncoupler induces mild ROS production and PQQ extends the lifespan by increasing endogenous ROS levels^89^. Quercetin, one of the antioxidant polyphenols, is also known to protect neural and cardiac tissues through its mild mitochondrial uncoupling function^90–92^. ROS can act as essential signaling molecules to promote metabolic health and longevity^93^. The mild mitochondrial uncoupling effect and the mild ROS production could be key factors in the protective pathway of PQQ. Based on these previous reports and the results in this study, we consider that the decrease in MMP dissipation at high doses of PQQ might be related to decreased cellular ATP levels and the enhanced regulation of ROS relating to apoptosis^44^. Previous reports have also suggested that the mitochondrial membrane permeability transition is a critical step in the induction of intrinsic apoptosis and often represents the earliest apoptotic signal^94^, and that the loss of mitochondrial ATP synthesis in apoptotic cells and the increase of inter-membrane creatine phosphate concentrations might be consequences of the loss of MMP caused by the translocation of proapoptotic BH3-only proteins to the mitochondria^95^. Because ROS can alter the expression level of many miRNAs^96^ and SIRT1 expression is also regulated by the miRNA pathway in oxidative stress^60^, ROS regulation of the uncoupling effect of PQQ can work protectively, through the miRNA post-translational modification pathway, in the oxidative stress-induced premature senescence of auditory cells.

This study demonstrated that PQQ protected mitochondrial function in HEI-OC1 auditory cells with stress-induced premature cellular senescence. The cell numbers and cell viability were protected from H_2_O_2_-induced cellular senescence by PQQ, which attenuated both H_2_O_2_-induced mitochondrial respiration decreases, as well as H_2_O_2_-induced morphological and dynamic movement changes. The mechanism underlying these findings may be associated with SIRT1, PGC-1α, and deacetylation of PGC-1α.

## Methods

### Cell culture and culture conditions

House Ear Institute-Organ of Corti 1 (HEI-OC1) cells derived from the auditory organ of the transgenic mouse Immortomouse^TM^, which harbors a temperature-sensitive mutant of the SV40 large T antigen gene under the control of an interferon-γ-inducible promoter element. HEI-OC1 cells represent a common progenitor for sensory and supporting cells of the organ of Corti^97,98^. HEI-OC1 cells provided by Professor F. Kalinec (UCLA, Los Angeles, CA, USA), were cultured in high-glucose Dulbecco’s Eagle’s medium (DMEM) (21063; Life Technologies, Inc, NY., USA) containing 10% fetal bovine serum (FBS) (Life Technologies, Inc, NY., USA) with 0.06% w/v penicillin (Nacalai Tesque, Kyoto, Japan). Cells were incubated at 33 °C with 10% CO_2_ under permissive conditions.

### PQQ treatments, cell viability assay and population doubling rate analysis

The HEI-OC1 auditory cells were incubated with PQQ (pyrroloquinoline quinone disodium salt, Mitsubishi Gas Chemical Co., Inc., Tokyo, Japan) in the culture medium for 1 day before H_2_O_2_ exposure. The HEI-OC1 auditory cells (4 × 10^4^ cells/mL/well of 12-well plates) were washed with Dulbecco’s phosphate-buffered saline (DPBS) once, then incubated with H_2_O_2_ (100 μM; Nacalai Tesque, Kyoto, Japan) dissolved in the DMEM without FBS for 1 h. The H_2_O_2_ solution was then replaced with the normal culture medium. The control cells were prepared in the same way, but without H_2_O_2_.

In the analysis of the cell population doubling rate and the cell viability, the cells were washed with DPBS, harvested from the flasks via trypsinization (0.05% trypsin, 0.53 mM EDTA for 2 min), and diluted (1:1) in 0.4% trypan blue solution. The population doubling rate and the cell viability for HEI-OC1 cells were measured using an image-based cell counter (Countess II; Thermo Fisher Scientific Inc., USA), following the manufacturer’s suggested procedure.

The metabolic activity of the HEI-OC1 cells after PQQ pretreatment was evaluated using the WST-8 (2-[2-methoxy-4-nitrophenyl]-3-[4-nitrophenyl]-5-[2,4-isulfophenyl]-2H-tetrazolium, monosodium salt) formazan assay. Cells were plated in a 96-well plate and cultured under multiple concentrations of PQQ in the medium for 1 day. Then the medium with PQQ was replaced with the normal culture medium and assays were performed using the Cell Counting Kit-8 (WST-8) (Dojindo, Kumamoto, Japan) by incubating cells with WST-8 for 1 hr as directed by the manufacturer and measuring the absorbance at 450 nm using the infinite M200 PRO-plate-reader (TECAN, USA). The mitochondrial membrane potential (MMP) is essential for maintaining mitochondrial biogenesis, and its disruption leads to cell apoptosis. To verify PQQ function with respect to the MMP, we utilized 5, 5’, 6, 6’-tetrachloro-1, 3,3’-tetraethyl benzimidazolyl carbocyanine iodide (JC-1) mitochondrial membrane fluorescent dye in analyzing the disruption of the MMP. JC-1 is taken up by the mitochondria and forms either JC-1 dimer aggregates with emission of red fluorescence (590 ± 17.5 nm) under high-membrane potentials, or JC-1 monomers with emission of green fluorescence (530 ± 15 nm) for low membrane potentials. The HEI-OC1 cells were exposed to multiple concentrations of PQQ which was then replaced with the normal medium with JC-1 (200 nM, Biotium, USA) for 30 min before the MMP ratio assay. The fluorescence emission ratio was analyzed with the infinite M200 PRO-plate-reader (TECAN, USA), under 10% CO_2_ incubation at 33 °C. The analysis of the red to the green ratio of JC-1 emissions was performed and calculated at multiple reads per well (circle, 4 × 4).

### Measurement of cellular senescence biomarkers

The cellular senescence biomarkers of the HEI-OC1 cells were evaluated with SA-β-Gal assay using SPiDER-βGal (SG02, DOJINDO, Japan) and CD26 (dipeptidyl peptidase-4 (DPP4)) (PE anti-mouse CD26, #137804, BioLegend, USA). In the analysis with SPiDER-βGal, the cells were washed with DPBS, harvested from the flasks via trypsinization (0.05% trypsin, 0.53 mM EDTA for 2 min), fixed with 4% paraformaldehyde in phosphate-buffered saline (PBS) (pH 7.4) (10010023; Thermo Fisher Scientific Inc., USA) for 3 min, washed with Hanks’ balanced salt solution (HBSS) with Ca and Mg without Phenol Red (09735-75; Nacalai Tesque, Kyoto, Japan) three times, incubated in 0.5 μM SPiDER-βGal in McIlvaine buffer (pH 6.0) diluted five times with distilled water at 37 °C in room air for 30 min, washed with HBSS two times and diluted in HBSS for flow cytometry analysis. In the analysis of CD26, the cells were washed with DPBS, harvested from the flasks via trypsinization (0.05% trypsin, 0.53 mM EDTA for 2 min), washed with PBS two times, stained with 1 μg/100 μL CD26 antibody in PBS with 0.1% bovine serum albumin (BSA) and 0.1% NaN_3_, washed with PBS three times and diluted in HBSS for flow cytometry analysis.

### Flow cytometry analysis

In the staining of SIRT1, the cells were washed with DPBS, harvested from the flasks via trypsinization (0.05% trypsin, 0.53 mM EDTA for 2 min), fixed with 2% paraformaldehyde in phosphate-buffered saline (PBS) (pH 7.4) (10010023; Thermo Fisher Scientific Inc., USA) at room temperature for 10 min, washed with PBS, incubated in 0.1% Triton X-100 in PBS at room temperature for 5 min, washed with PBS two times, stained with Alexa Fluor 488 Phalloidin (12:1000, A12379; Thermo Fisher Scientific Inc., USA) and anti-SIRT1 antibody (1:500, NBP1-51641AF647 (1.77 mg/mL), Novus Biologicals, Inc.) in PBS with 0.5% BSA at room temperature for 30 min, washed with PBS two times and diluted in PBS for flow cytometry analysis.

In the staining of MitoSOX Red, the cells were washed with DPBS, harvested from the flasks via trypsinization (0.05% trypsin, 0.53 mM EDTA for 2 min), washed with HBSS 2 times, stained with MitoSOX Red (5 µM, Thermo Fisher Scientific Inc., USA) in HBSS at 37 °C in room air for 10 min, washed with HBSS two times and diluted in HBSS for flow cytometry analysis.

The distribution of fluorescent intensity in HEI-OC1 cells was analyzed using a multi-color flow cytometer (Gallios, Beckman Coulter, Brea, CA, USA). The staining of both SPiDER-βGal, CD26 and MitoSOX Red was filtered with a 40 μm cell strainer and were measured using FL2 detector (488 nm excitation, 575/20 nm emission). The staining of SIRT1 was measured using FL6 detector (633 nm excitation, 660/20 nm emission). The analysis was performed using Flowing Software (Turku Bioscience Centre, Finland), flowCore^99^, and Bioconductor^100^.

### Measurement of mitochondrial function using the XF24 extracellular flux analyzer

For the further protective analysis, four groups were determined: (1) control group, no PQQ treatment; (2) H_2_O_2_ group, treated with 100 μM H_2_O_2_ for 1 h; (3) 0.1 nM PQQ group, pretreatment with 0.1 nM PQQ for 1 day followed by 100 μM H_2_O_2_ for 1 h; 4) 1.0 nM PQQ group, pretreatment with 1.0 nM PQQ for 1 day followed by 100 μM H_2_O_2_ for 1 h. Cells were cultured in XF24 plate, pretreated with PQQ for 1 day followed by 100 μM H_2_O_2_ for 1 h. A Seahorse XF24 Analyzer (Agilent Technologies, Santa Clara, CA) was used to measure mitochondrial function in HEI-OC1 cells. The cells in each of the four groups as listed above were rinsed twice with assay medium (XF assay medium (DMEM without NaHCO_3_, without GlutaMAX, without l-glutamine; 102353-100, Seahorse Bioscience, Billerica, MA, USA, Supplementary Data), supplemented with 25 mM (450 mg/dL) d-glucose (Otsuka Seiyaku, Tokushima, Japan) and 1 mM sodium pyruvate (Nacalai Tesque, Kyoto, Japan)) and then equilibrated for 1 hr at 33 °C in an incubator (0.03% CO_2_) prior to the assay. The mitochondrial basal respiration was measured before any pharmacological perturbations. The cells were then subjected to (in sequence, in final concentrations): (1) 2.5 μM Oligomycin A to inhibit ATP synthase and oxidative phosphorylation (OXPHOS); (2) 1.25 μM carbonyl cyanide-4-(trifluoromethoxy) phenylhydrazone (FCCP) to induce maximal respiration; (3) 0.625 μM Rotenone; (4) 0.625 μM Antimycin A to end this reaction under assay medium. The XF24 analyzer determines the oxygen and proton concentrations in real time which reflects the rate of oxygen consumption and proton production speeds. Parameters expressed as the oxygen consumption rate (OCR) in pMoles/min or the extracellular acidification rate (ECAR) in mpH/min were key indicators of mitochondrial respiration and glycolysis. The ATP production rates were calculated according to the manufacturer’s instructions and software^101,102^. In the substrate oxidation stress test, the cells were prepared as above prior to the assay, and the cells were subjected to (in sequence, in final concentrations): (1) 0, 4 mM l-glutamine (Seahorse Bioscience, Billerica, MA, USA), 40 μM Etomoxir (11969, Cayman Chemical, Ann Arbor, USA) or 3 μM BPTES ((bis-2-(5-phenylacetamido-1,3,4-thiadiazol-2-yl)ethyl sulfide); 19284, Cayman Chemical, Ann Arbor, USA) (2) 5 μM Oligomycin A; (3) 2.5 μM FCCP. In the fatty acid oxidation (FAO) assay, the culture medium was washed twice and replaced with substrate-limited DMEM (102353-100, Seahorse Bioscience, Billerica, MA, USA) supplemented with 25 mM d-glucose, 1 mM sodium pyruvate, 0.5 mM l-carnitine hydrochloride (07353-51, Nacalai Tesque, Kyoto, Japan) and 0.5 mM NaOH to adjust to pH 7.4, and the cells were equilibrated for 1 h at 33 °C in an incubator (0.03% CO_2_) prior to the assay. In the FAO assay, the cells were subjected to (in sequence, in final concentrations): (1) conjugate of palmitatic acid (PA) (25919-62, Nacalai Tesque, Kyoto, Japan) and fatty acid-free bovine serum albumin (BSA) (08587-26, Nacalai Tesque, Kyoto, Japan) (final concentration PA: 200 µM, BSA 34 µM) or BSA (34 µM) only (2) 5 μM Oligomycin A; (3) 2.5 μM FCCP. The number of cells was counted after each XF24 analysis as follows; the cells in the XF24 plate was washed with PBS 1 time, fixed with 4% paraformaldehyde (PFA) (Nacalai Tesque, Kyoto, Japan) for 1 min, stained with Hoechst 33342 (final 5 µg/mL, Thermo Fisher Scientific Inc., USA) for 30 min, washed with PBS one time, captured with a fluorescence microscope (BZ-X710, KEYENCE, Osaka, Japan) using the DAPI filter cube, and counted automatically using ImageJ^103,104^ with Fiji^105^.

### Analysis of mitochondrial dynamics and morphology

Cells were dyed with tetramethylrhodamine, ethyl ester (TMRE) (200 nM, Biotium, USA) mitochondrial fluorescence and captured with confocal microscopy focusing on mitochondrial-rich regions beside the cell nucleus. Z stacks across the depth of the cell provide 3D information about mitochondrial morphology while the time-lapse images allow the study of changes in mitochondrial dynamics over time. Images were captured at 3 s of intervals for a duration of 3 min. Supplementary Video 1 depicts a representation of a projection of Z stacks of mitochondria in a single cell with a focus on the mitochondrial-rich regions. All image processing and analysis were performed using NIS-Elements (Nikon, Japan), ImageJ^103,104^ with Fiji^105^, TrackMate^106^, and @msdanalyzer^107^ in MATLAB R2019b. The mean square displacement (MSD) and the area under curve (AUC) measurements were used for the analysis of the parameters of mitochondrial motion. The mitochondrial skeleton was vectorized to measure branches, junctions, and average branch lengths as described in our previous study^5^.

### Western blot

The cells were washed three times with ice-cold PBS and then lysed in RIPA lysis buffer containing protease inhibitor cocktails (in final concentrations; 10 mM NaF, 1 mM Sodium orthovanadate, 1 mM PMSF, 1× cOmplete Mini (Roche, Mannheim, Germany), 0.3 µg/mL Trichostatin A, 10 mM Nicotinamide, 5 mM Beta-glycerophosphoric acid, 1 mM dithiothreitol (DTT)) for 30 min on ice. Insoluble fractions of cell lysates (2–3 mg) were removed by centrifugation at 13,000 ×*g* at 4 °C for 5 min and denatured by boiling in EzApply (AE-1430; ATTO Co, Japan). The protein concentrations were determined using a Pierce BCA Protein Assay Kit (Thermo Fisher Scientific, Inc., Waltham, MA, USA). The samples containing 60 μg of proteins were separated by SDS-PAGE using EzRunC + (AE-1412; ATTO Co, Japan) and transferred onto PVDF membranes using the BioRad TransBlot Turbo according to the manufacturer’s instructions (High MW 10 min for SIRT1, Mixed MW 7 min for PGC-1α and Ac-Lysine). Following blocking with 5% nonfat milk (Amersham ECL blocking agent, GE Healthcare) at room temperature for 1 hr, membranes were incubated with primary antibodies at 4 °C overnight and then incubated with horseradish peroxidase (HRP) conjugated secondary antibodies (anti-mouse 1:200,000, ab6789, Abcam; anti-rabbit 1:200,000, NA934VS, Sigma Aldrich) for 1 h at room temperature. The detection was carried out using the chemiluminescent imaging system (ImageQuant LAS 4010, UK) after incubation with SuperSignal^TM^ West Femto Maximum Sensitivity Substrate (Thermo, USA) according to the manufacturer’s instructions. The primary antibodies used were SIRT1 (1:500, (B-7) sc-74465, Santa Cruz), PGC-1α (1:200, (D-5) sc-518025, Santa Cruz), and β-actin (1:10,000, PM053, MBL).

All blots derive from the same experiment and were processed in parallel.

### Immunoprecipitation and PGC-1α acetylation

Immunoprecipitation was performed according to the manufacturer’s directions (Thermo Fisher Scientific, Inc., Waltham, MA, USA): 50 μL Dynabeads Protein G was added to the 500 μg of sample solution and shaken on a horizontal shaker for 10 min to eliminate nonspecific binding proteins before separating on the magnet until the supernatant was clear and could be removed. 4 μg primary antibody against PGC-1α and 50 μL of protein G agarose were then added into the supernatant and shaken on a horizontal shaker for 10 min at room temperature. After washing three times, the beads were eluted with 50 mM Glycine (pH 2.8) for 2 min and then separated with SAS-PAGE as described in the western blot protocol above. The membranes were incubated with acetyl-lysine HRP (1:200, (7F8) sc-81623 HRP, Santa Cruz) to stain at 4 °C overnight after blocking the transferred membrane. The membranes were then detected with the same procedure described above before being reproved with anti-PGC-1α HRP (1:200, (D-5) sc-518025 HRP, Santa Cruz) antibody and a secondary antibody (anti-mouse 1:1000 Mouse TrueBlot ULTRA, 18-8817-30, Rockland).

### Quantitative RT-PCR analysis

Total RNA was isolated with NucleoSpin RNA (Macherey-Nagel, Düren, Germany) according to the manufacturer’s instructions. The concentration of extracted mRNA was measured using NanoDrop Lite (Thermo Fisher Scientific, USA) and complementary DNA (cDNA) was generated using ReverTraAce qPCR RT Master Mix with gDNA Remover (TOYOBO, Japan) according to the manufacturer’s instructions. The quality of mRNA was assessed by visualization of the 28S and 18S ribosomal RNA bands using DynaMarker, RNA High for Easy Electrophoresis (BioDynamics Laboratory Inc., Japan). The primers of genes including SIRT1 (forward GCTGACGACTTCGACGACG, reverse TCGGTCAACAGGAGGTTGTCT, final concentration 250 nM), PGC-1α (forward AAGTGGTGTAGCGACCAATCG, reverse AATGAGGGCAATCCGTCTTCA, final concentration 50 nM), Cdkn2a (p16) (forward CGCAGGTTCTTGGTCACTGT, reverse TGTTCACGAAAGCCAGAGCG, final concentration 500 nM), Cdkn1a (p21) (forward CCTGGTGATGTCCGACCTG, reverse CCATGAGCGCATCGCAATC, final concentration 500 nM), Trp53 (p53) (forward GCGTAAACGCTTCGAGATGTT, reverse TTTTTATGGCGGGAAGTAGACTG, final concentration 500 nM), Cpt1a (forward GTCCCTCCAGCTGGCTTATC, reverse CATGCGGCCAGTGGTGTCTA, final concentration 1000 nM), Cpt1c (forward TCTTCACTGAGTTCCGATGGG, reverse ACGCCAGAGATGCCTTTTCC, final concentration 125 nM), Cpt2 (forward CCCTGCATACCAGCGGATAA, reverse CATACGCAATGCCAAAGCCA, final concentration 500 nM), Gls1 (forward TTCGCCCTCGGAGATCCTAC, reverse CCAAGCTAGGTAACAGACCCT, final concentration 500 nM), Gls2 (forward CGTCCGGTACTACCTCGGT, reverse TGTCCCTCTGCAATAGTGTAGAA, final concentration 250 nM), GAPDH (forward TGAGGCCGGTGCTGAGTATGTCG, reverse CCACAGTCTTCTGGGTGGCAGTG, final concentration 125 nM) and β-actin (forward CCTCTATGCCAACACAGTGC, reverse GTACTCCTGCTTGCTGATCC, final concentration 250 nM) were purchased from FASMAC (Kanagawa, Japan). The forward and reverse primers were mixed with 100 ng cDNA and quantitative RT-PCR was performed using PowerUp SYBR Green Master Mix (Applied Biosystems, Darmstadt, Germany) or THUNDERBIRD SYBR qPCR Mix (TOYOBO, Japan). The following experimental run protocol was used: denaturation and activation program (50 °C for 120 s, 95 °C for 120 s), amplification and quantification program repeated 60 times (95 °C for 15 s, 60 °C for 60 s), melting curve program (95 °C for 15 s, 60 °C for 60 s, 95 °C for 15 s). Data collection was performed using QuantStudio 7 Flex Detection System (Thermo Fisher Scientific, USA). The 2-ΔΔCt method was applied to analyze the relative changes in gene expressions. The mRNA expression level of each gene was normalized using β-actin as an internal control.

### Mitochondrial DNA copy number analysis

Total DNA was isolated with NucleoSpin Tissue (Macherey-Nagel, Düren, Germany) according to the manufacturer’s instructions. The concentration of extracted DNA was measured using NanoDrop Lite (Thermo Fisher Scientific, USA). The primers of mitochondrial and nuclear genes including mitochondrial 16 S rRNA (forward CCGCAAGGGAAAGATGAAAGAC, reverse TCGTTTGGTTTCGGGGTTTC, final concentration 1000 nM), mitochondrial NADH-ubiquinone oxidoreductase chain 1 (ND1) (forward CTAGCAGAAACAAACCGGGC, reverse CCGGCTGCGTATTCTACGTT, final concentration 500 nM), mitochondrial D-loop (forward CCAAAAAACACTAAGAACTTGAAAGACA, reverse GTCATATTTTGGGAACTACTAGAATTGATC, final concentration 1000 nM), nuclear hexokinase 2 (HK2) (forward GCCAGCCTCTCCTGATTTTAGTGT, reverse GGGAACACAAAAGACCTCTTCTGG, final concentration 500 nM) and nuclear thymidine kinase 1 (TK1) (forward GACTGTATTGAGCGGCTTCAGA, reverse CATGCTCGGTGTGAGCCATA, final concentration 500 nM) were purchased from FASMAC (Kanagawa, Japan). Quantitative PCR was performed in the same way as in quantitative RT-PCR analysis. The copy number of mitochondrial DNA was calculated using the deviations of the cycle threshold (Ct) between mitochondrial and nuclear genes according to the previous studies^108,109^.

### Measurement of glucose concentration of the medium

The glucose concentration of the medium was measured using the following glucometers: Accu-Chek Aviva Nano/Guide (Roche Diagnostics GmbH, Mannheim, Germany), OneTouch Verio Reflect (Johnson & Johnson, New Jersey, USA), Freestyle Libre (Abbott Diabetes Care, Witney, U.K.) and NIPRO StatStrip XP2 (NIPRO, Osaka, Japan).

### Statistical analysis

The statistical software GraphPad Prism and R version 4.1.0 software (R Core Team; R Foundation for Statistical Computing, Vienna, Austria, 2021) were used in data processing and statistical analysis. A one-way ANOVA with Bonferroni’s test was used to make multiple comparisons between groups. A value of *P* < 0.05 was considered significant. Box plot shows statistical parameters as follows; central line: median; box limits: first and third quartile; whiskers: minimum and maximum.

### Reporting summary

Further information on research design is available in the Nature Research Reporting Summary linked to this article.

## Supplementary information

Video 1

Supplementary Data

Reporting Summary

## Data Availability

Data are available from the corresponding author on a reasonable request.
