# Author Correction: Pyrroloquinoline quinone (PQQ) protects mitochondrial function of HEI-OC1 cells under premature senescence

**DOI:** 10.1038/s41514-022-00095-w

**Published:** 2022-10-06

**Authors:** Ying Gao, Teru Kamogashira, Chisato Fujimoto, Shinichi Iwasaki, Tatsuya Yamasoba

**Affiliations:** 1 Department of Otolaryngology and Head and Neck Surgery, Faculty of Medicine, University of Tokyo, Tokyo, Japan; 2 Department of Otolaryngology and Head and Neck Surgery, The Second Affiliated Hospital of Xi’an Jiaotong University, Xi’an, China; 3 Department of Otolaryngology & Head and Neck Surgery, Nagoya City University Graduate School of Medicine, Nagoya, Japan

**Keywords:** Senescence, Organelles

Correction to: *npj Aging* 10.1038/s41514-022-00083-0, published online 19 April 2022

In the original version of this Article, the text “This work was supported by JSPS KAKENHI Grant Numbers 25293347, 26253081, 18K16906, 18K19602, 20H00546, 20K21646 and 21K16853” was mistakenly left out of the Acknowledgements. The HTML and PDF versions of this Article have now been corrected.

