# Supplementary material for: Pyrroloquinoline quinone (PQQ) protects mitochondrial function of HEI-OC1 cells under premature senescence
Source: NPJ Aging. 2022 Apr 19;8(1):3. doi: 10.1038/s41514-022-00083-0 (PMC9158787; doi:10.1038/s41514-022-00083-0)
Supplement: Supplementary file 2 — Supplementary Data [file 41514_2022_83_MOESM2_ESM.pdf]

Supplementary Information

Supplementary Figure 1 Full-length Western blot images

(a) Full-length blot image for SIRT1.

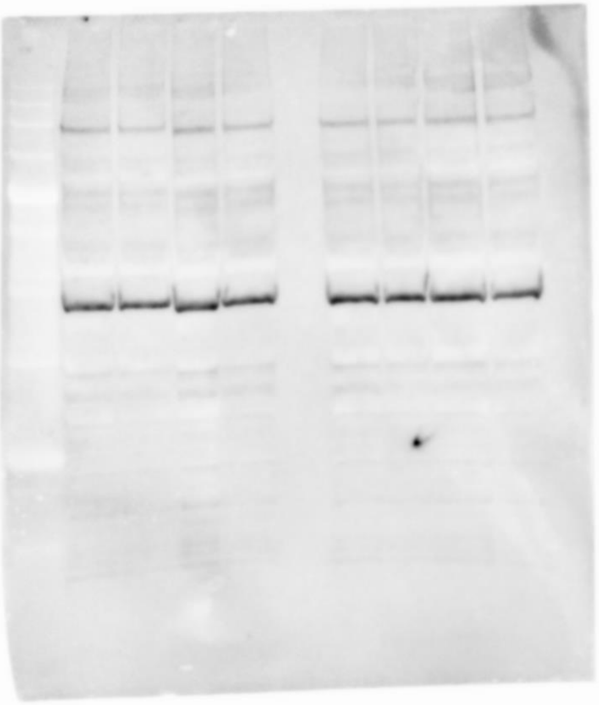

(b) Full-length blot image for PGC-1α.

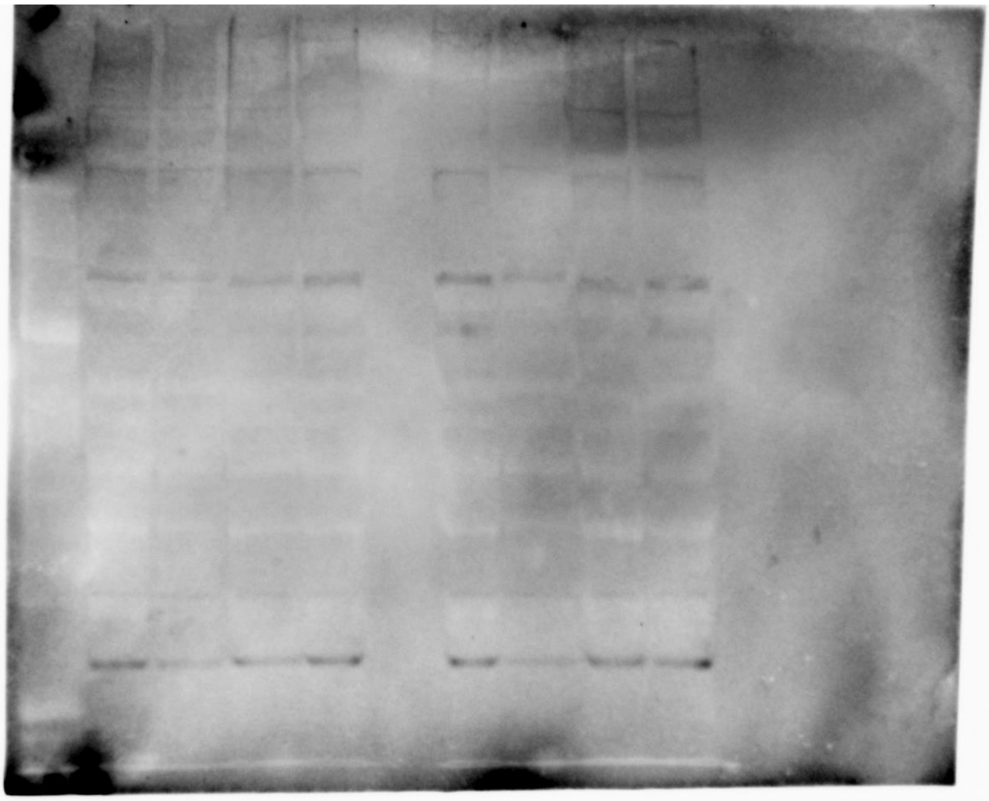

(c) Full-length blot image for β-actin.

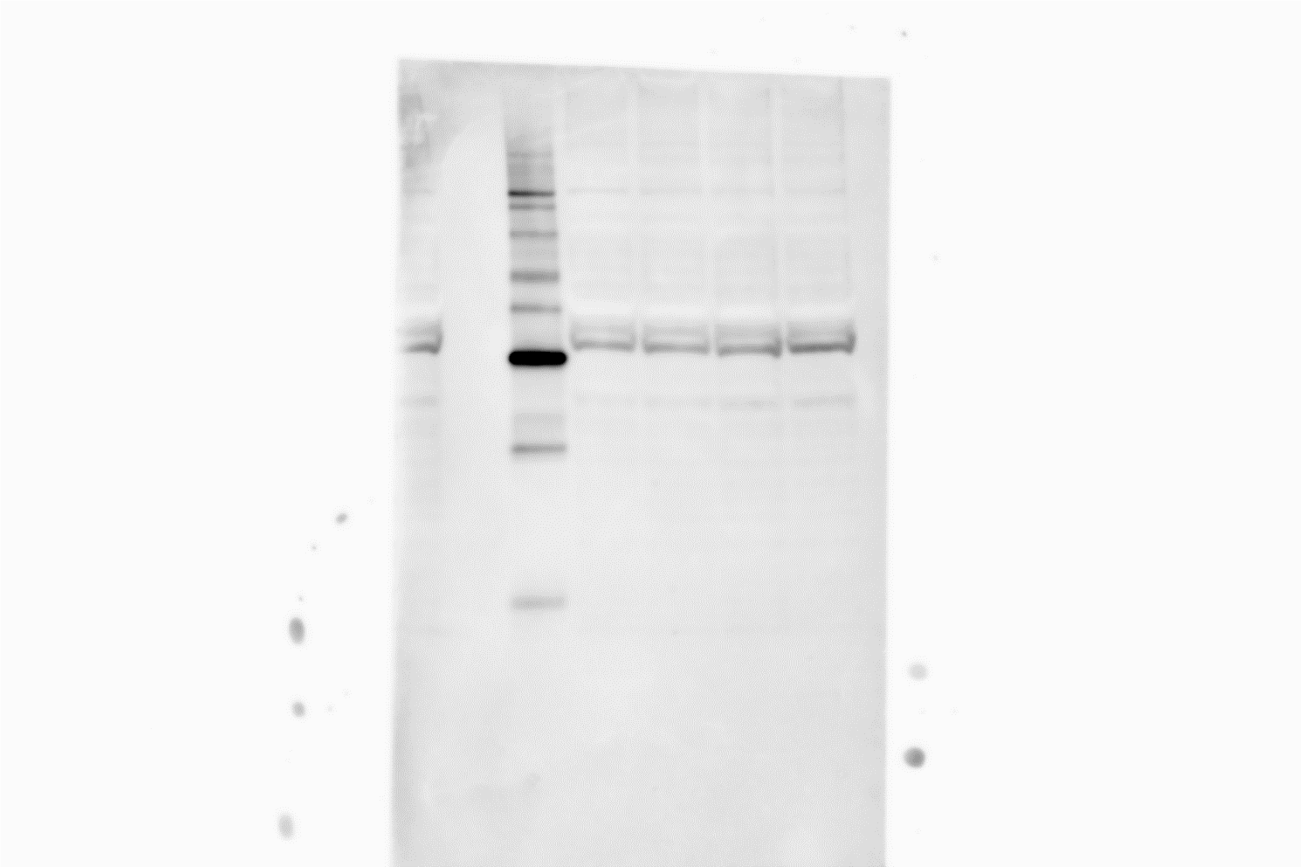

(d) Full-length blot image for acetylated-lysin (immunoprecipitation).

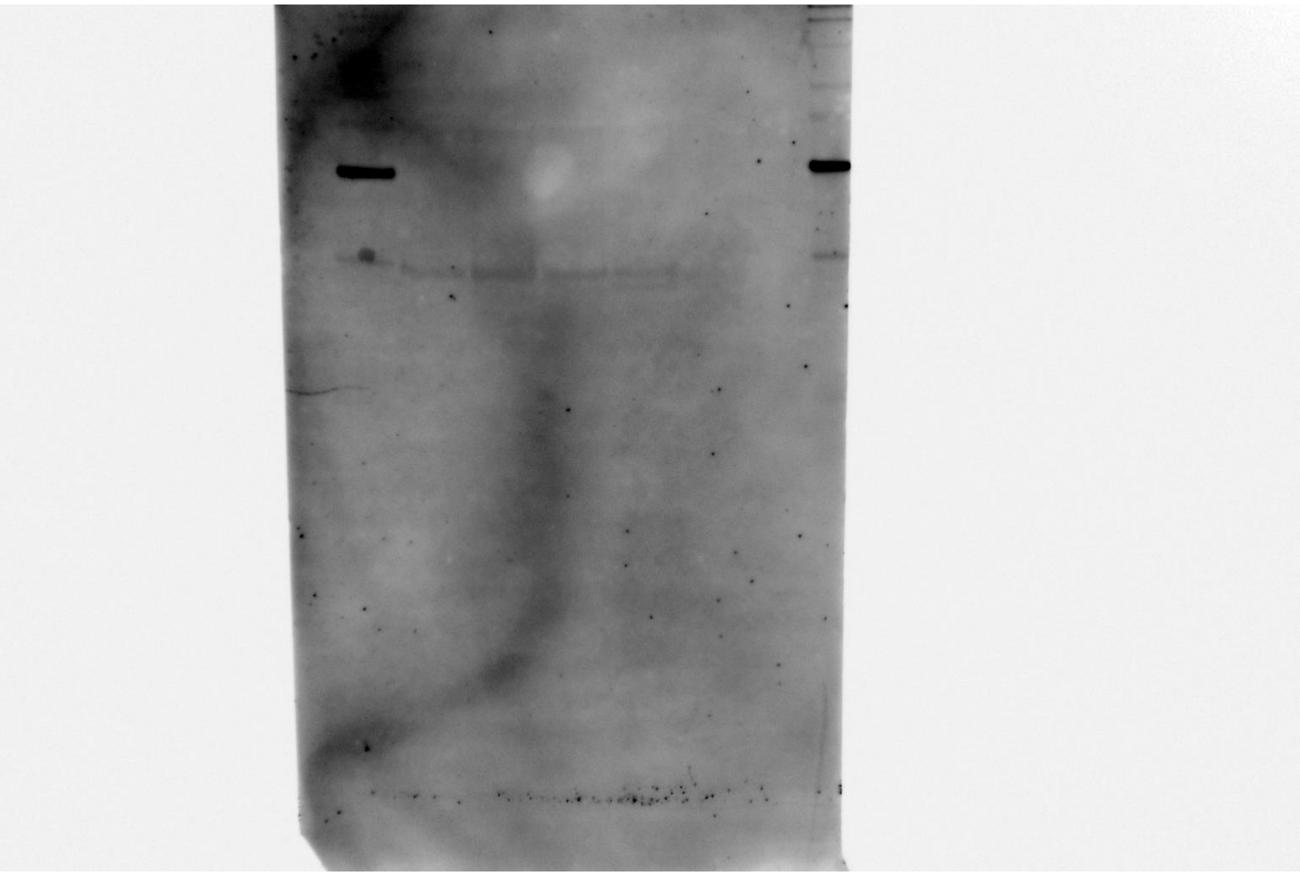

(e) Full-length blot image for PGC-1α (immunoprecipitation).

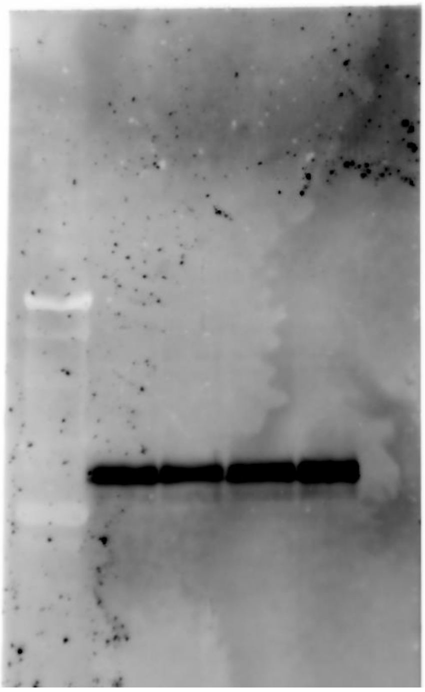

Supplementary Figure 2 SPiDER-βGAL microscopic images.

(a) Control group

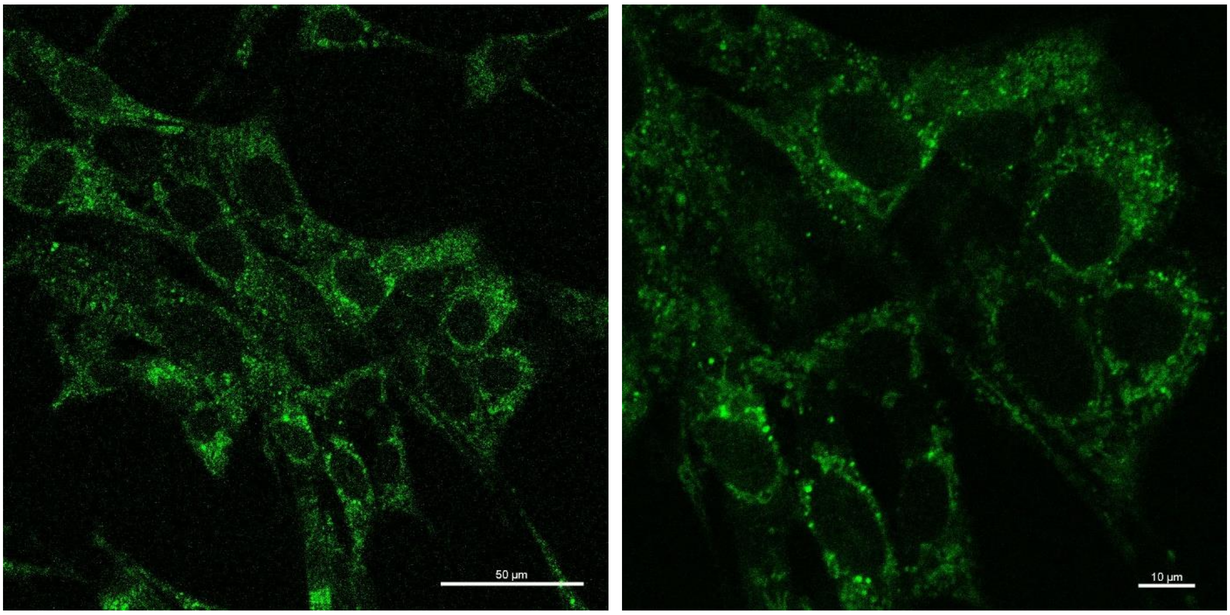

(b) H_2O_2 exposured-group

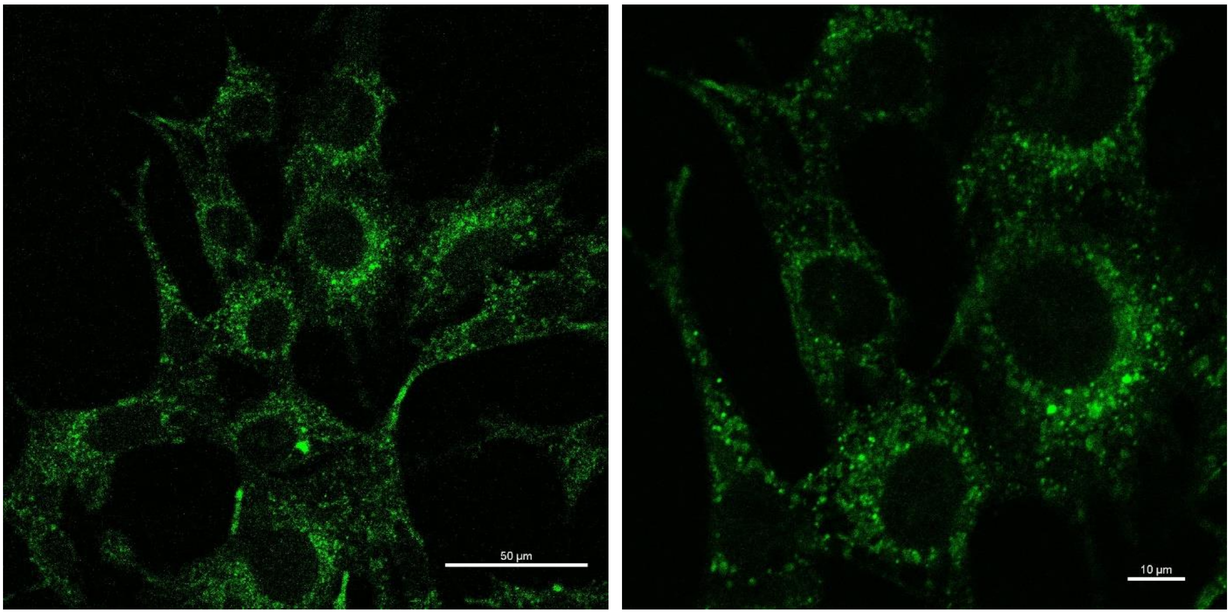

(c) PQQ 0.1nM treated H_2O_2 exposured-group

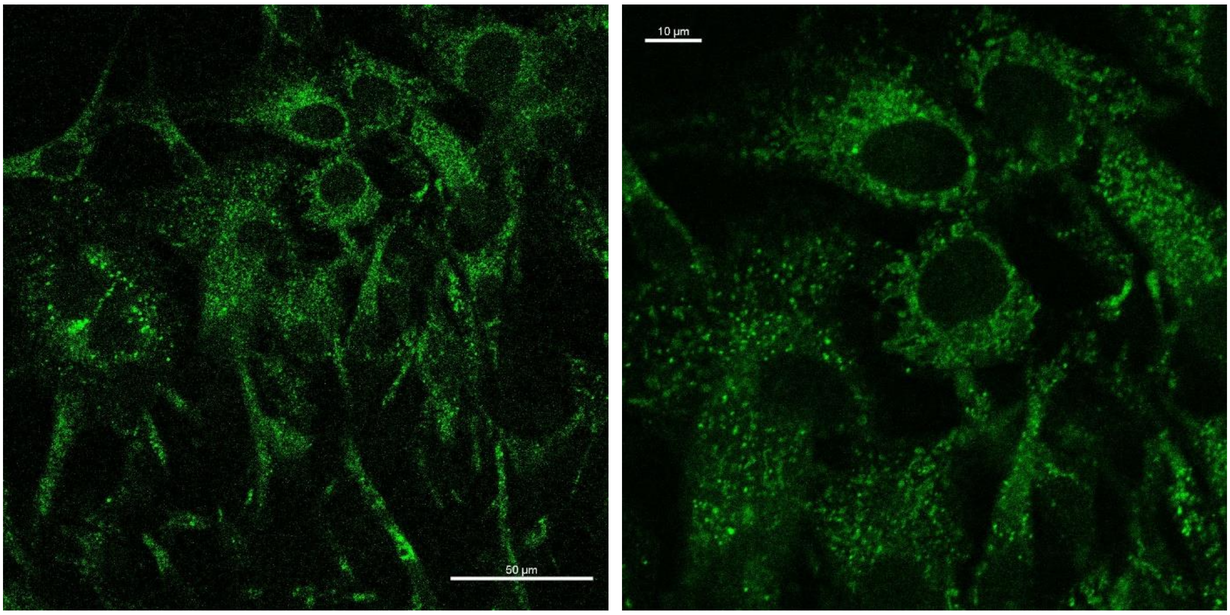

(d) PQQ 1.0nM treated H_2O_2 exposured-group

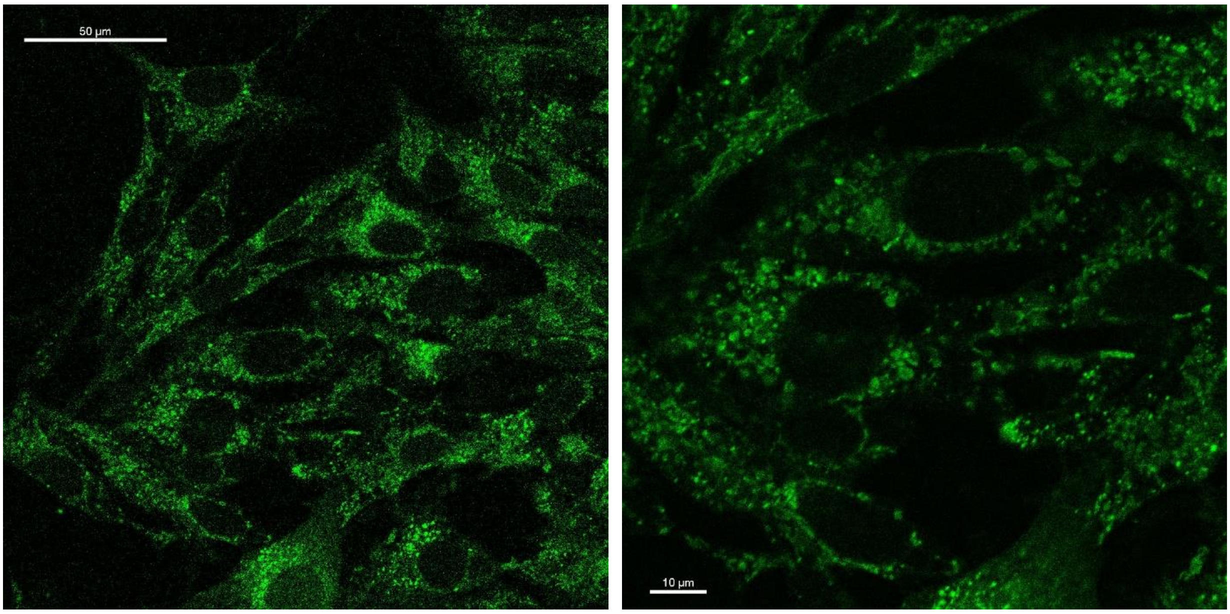

Supplementary Figure 3 Flowcytometry analysis of PQQ treatment using MitoSOX Red fluorescent dye

(a) The changes of MitoSOX Red intensity histogram in flow cytometry analysis.

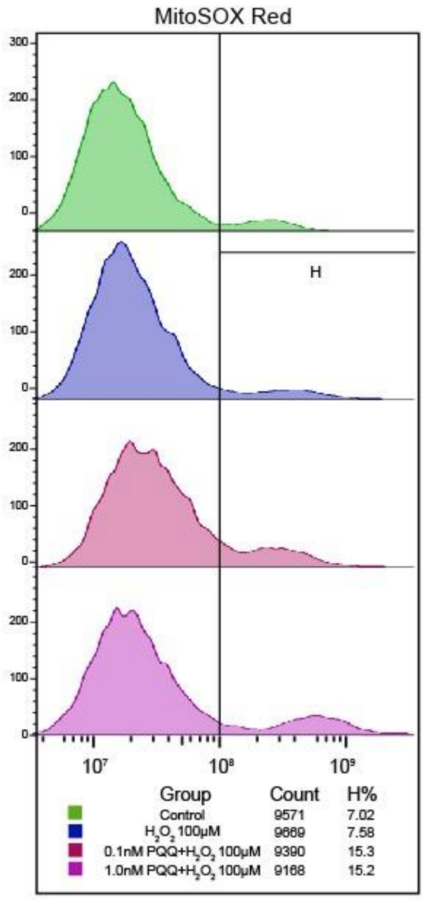

(b) The increased ratio in positivity rate over control of MitoSOX Red positive cells. (n = 5 per group; repeated experiments)

Box plot shows statistical parameters as follows; central line: median; box limits: first and third quartile; whiskers: minimum and maximum. * $p<0.05$.

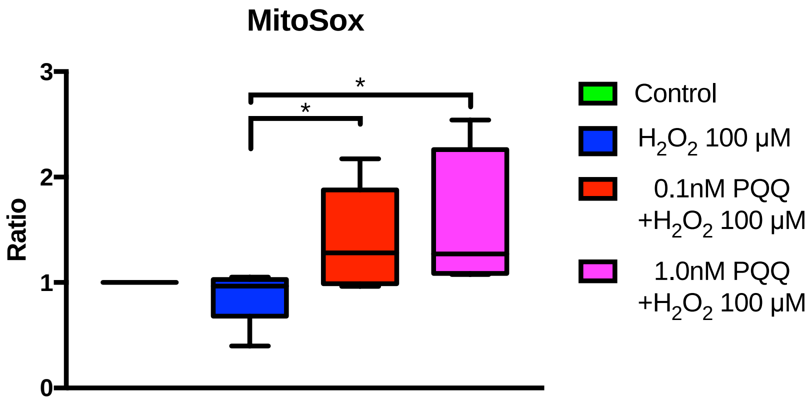

Supplementary Figure 4 The pH measurements of the XF24 analyzer

Data are shown as mean ± standard deviation.

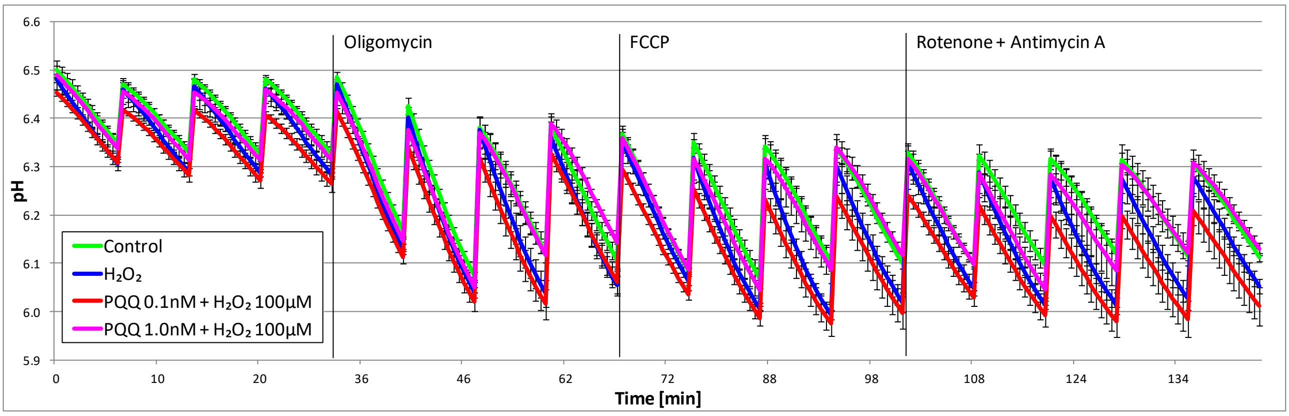

Supplementary Figure 5 The mitochondrial copy number analysis

Data are shown as mean ± standard deviation.

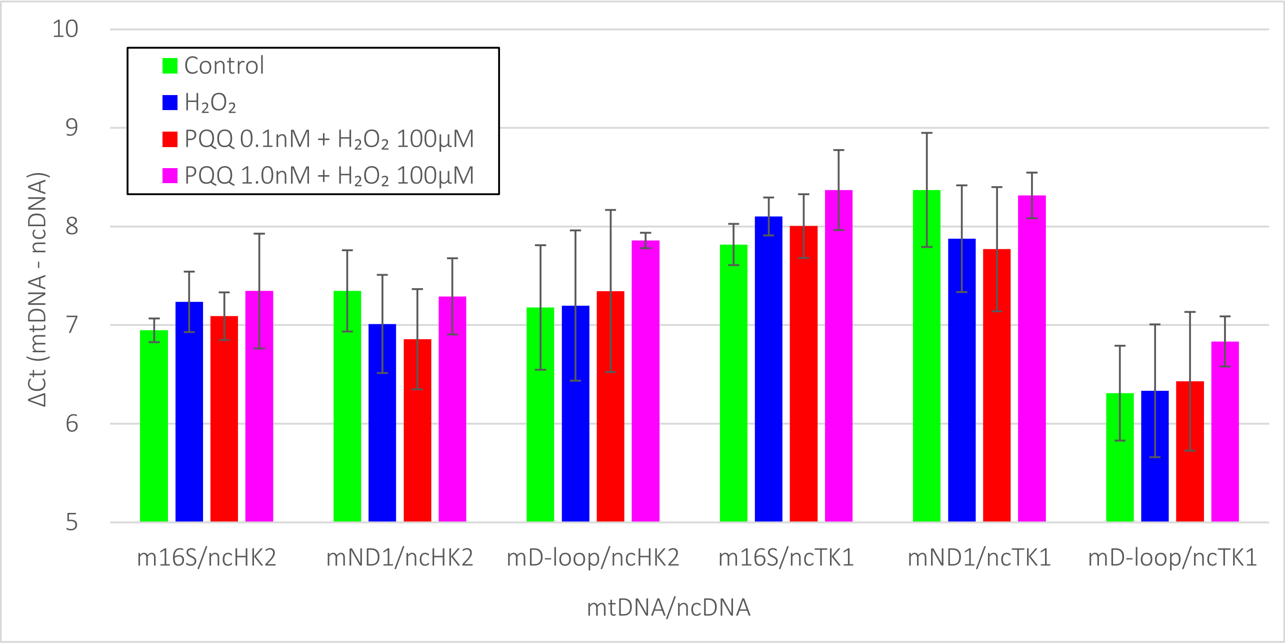

Supplementary Figure 6 Change in glucose concentration of the medium in the control group over time
The glucose concentration of the medium decreased overtime from the days of passages (2021/6/27 and 2021/7/2).

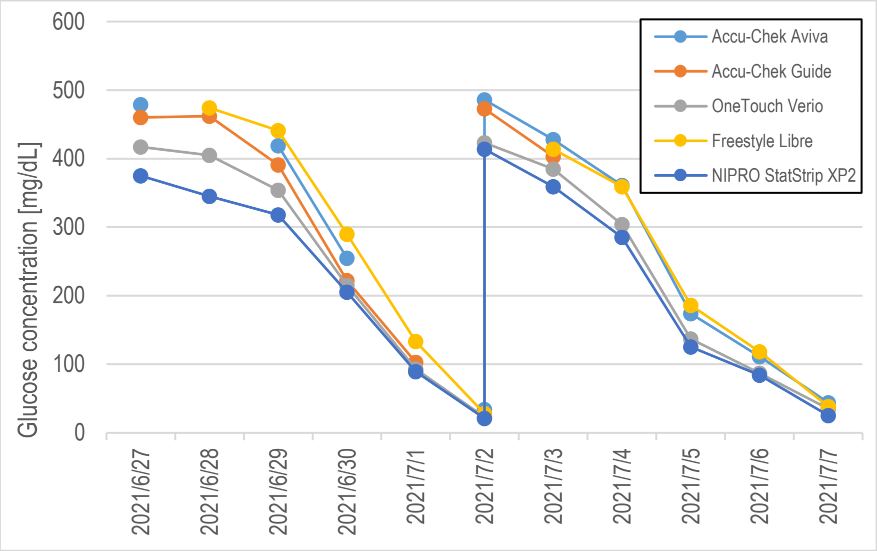

Supplementary Data 1 XF Base Medium Formulation

$MgSO_4$	0.8 mM
$CaCl_2$	1.8 mM
NaCl	143 mM
KCl	5.4 mM
NaH_2PO_4	0.91 mM
Phenol Red	3 mg/L

Other Constituents:

L-Arginine*HCl, L-Cystine*2HCl, Glycine, L-Histidine*HCl*H_2O, L-Isoleucine, L-Leucine, L-Lysine*HCl, L-Methionine, L-Phenylalanine, L-Serine, L-Threonine, L-Tryptophan, L-Valine, L-Tyrosine*2Na*2H_2O, Folic Acid, Riboflavin, D-Ca-Pantothenate, Choline Chloride, i-Inositol, Nicotinamide, Pyridoxine*HCl, Thiamine*HCl
